# Supplementary figures and images for: Oxytetracycline hyper-production through targeted genome reduction of Streptomyces rimosus
Source: mSystems. 2024 Apr 2;9(5):e00250-24. doi: 10.1128/msystems.00250-24 (PMC11097637; doi:10.1128/msystems.00250-24)

Legend: Clustering analysis of RNA-seq data with gene identifiers

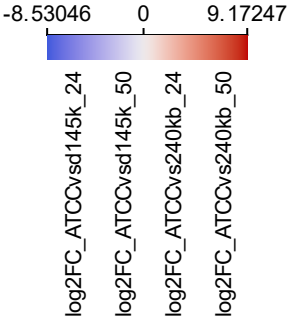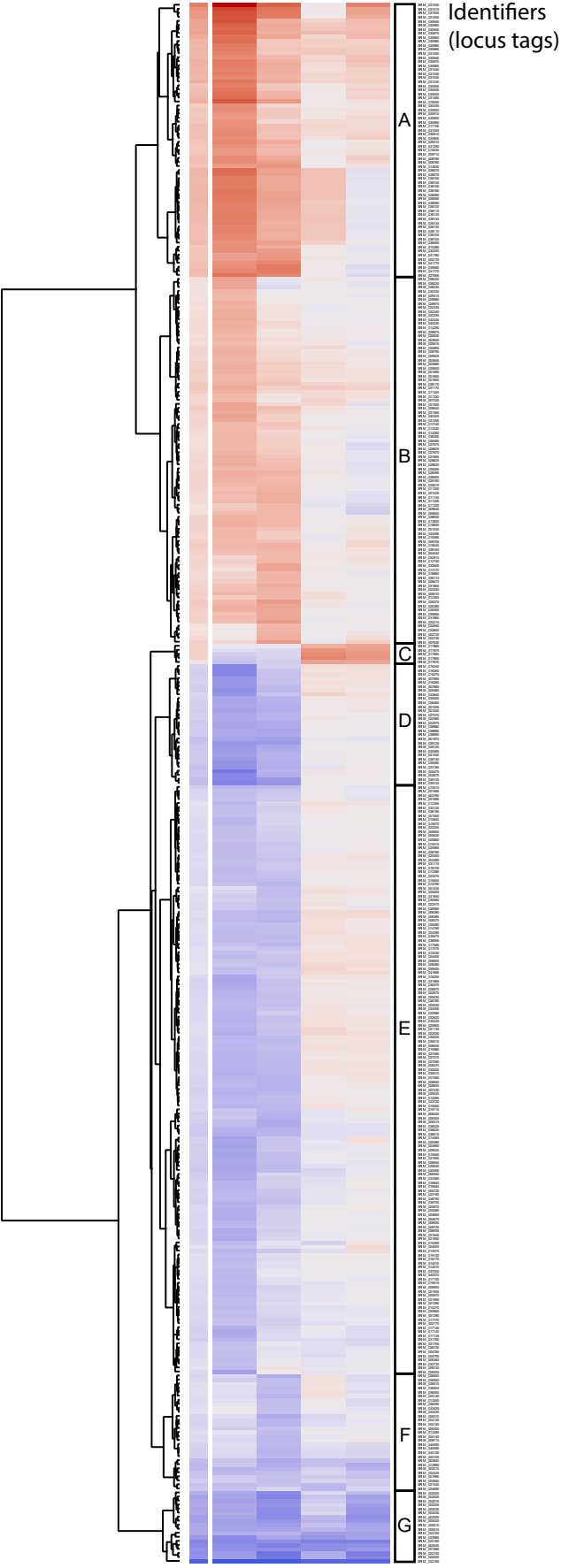

Supplement: Data S2 — Results of RNA-seq clustering analyis: heatmap (Fig. 5) with marked gene locus tags. [file msystems.00250-24-s0002.pdf]
