# Supplementary material for: Oxytetracycline hyper-production through targeted genome reduction of Streptomyces rimosus
Source: mSystems. 2024 Apr 2;9(5):e00250-24. doi: 10.1128/msystems.00250-24 (PMC11097637; doi:10.1128/msystems.00250-24)
Supplement: Additional experimental details — Supplemental information, figures, and tables. [file msystems.00250-24-s0003.docx]

**Supplementary information:**

Oxytetracycline hyper-production through targeted genome reduction of *Streptomyces rimosus*

Alen Pšeničnik^1^, Lucija Slemc^1^, Martina Avbelj^1^, Miha Tome^1*^, Martin Šala^4^, Paul Herron^5^, Maksym Shmatkov^3^, Marko Petek^2^, Špela Baebler^2^, Peter Mrak^6^, Daslav Hranueli^3^, Antonio Starčević^3^, Iain S. Hunter^5^, Hrvoje Petković^1^

**Affiliations:**

^1^University of Ljubljana Biotechnical Faculty, Chair of Biotechnology, Microbiology and Food Safety, Ljubljana, Slovenia

^2^National Institute of Biology, Ljubljana, Slovenia

^3^ University of Zagreb, Faculty of Food Technology and Biotechnology, Zagreb, Croatia

^4^National Institute of Chemistry, Ljubljana, Slovenia

^5^Strathclyde Institute of Pharmacy and Biomedical Sciences, University of Strathclyde, Glasgow G4 0RE, UK.

^6^Sandoz, Antiinfectives, Mengeš, Slovenia

*Current affiliation: National Institute of Biology, Ljubljana, Slovenia

**Supplementary information 1:**

**Assembly of the *Streptomyces rimosus* M4018 and R6-500 closed genome sequences**

The complete genome sequences of M4018 and R6-500 strains were assembled using a combination of long-read (LR) PacBio sequencing and short-read (SR) Illumina sequencing. LRs were first filtered by FiltLong (<https://github.com/rrwick/Filtlong>) (Galaxy Version 0.2.1+galaxy0) with ‘Min. length’ and ‘keep percentage’ set at 6000bp and 90%, respectively. This yielded 67,666 reads between 6000 and 43,024bp in length for M4018, whilst for R6-500 there were 37,152 reads between 6000 and 44,888bp in length. Meanwhile, SRs were polished with fastp [1] (Galaxy Version 0.23.2+galaxy0) using default settings that generated 2,044,872 reads with a mean coverage of 57x for M4018 and 15,291,349 reads and 256x mean coverage for R6-500, respectively. Unless otherwise stated, the software was hosted on Galaxy Europe [2] (<https://usegalaxy.eu/>) and default settings were used. LRs were first assembled using Canu Assembler [3] (Galaxy Version 2.1.1+galaxy0) and SRs were independently assembled using unicycler (Galaxy Version 0.5.0+galaxy1). For M4018, two LR contigs were obtained from Canu, one similar to the chromosome and another to the giant linear plasmid (GLP), pSRP1, of *S. rimosus* ATCC 10970. SR contig #301 overlapped with one end of LR chromosomal contig and allowed us to extend the chromosome by 642bp. Due to duplication at the other end of the chromosome (see later), it was not possible to extend the other end of this LR contig. The plasmid LR contig overlapped with SR contig #432 and 1bp was added to the plasmid, whilst 6,841bp of SR Contig #231 was added to the other end. For R6-500, three LR contigs were generated from Canu of 8,063,974; 541,869 and 185,135bp and were designated LR contigs A, B and C, respectively. Contigs A and B displayed similarity to the ATCC 10970 [22] chromosome and Contig C to pSRP1. The ends of Contig A overlapped with Contig B in two places, which suggested that Contig B represented a duplicated terminal inverted repeat. Consequently, we extended Contig A with 527,486bp of Contig B at one end and 526,185bp of Contig B at the other. We were then able to extend both ends of the R6-500 chromosome with 1312bp of SR contig #11. Contig C, the plasmid, was extended at each end by 8,696bp and 4,015bp of SR Contig #23 respectively. Finally, Bwa-Mem2 [73] (Galaxy Version 2.2.1+galaxy0) was used to generate BAM files of both replicons from each strain that were subsequently used to generate our final sequences with Pilon [74] (Galaxy Version 1.20.1), where the Minimum gap size was set at 100. The assemblies were then evaluated using Quast (8) (Galaxy Version 5.2.0+galaxy0) with ATCC 10970 set as a reference sequence that allowed us to display Circo’s versions of the Icarus contig alignment viewer mapped against PacBio reads for each strain. Annotations were carried out using NCBI Prokaryotic Genome Annotation Pipeline (PGAP) and are available as Bioproject PRJNA1031826 and Biosamples SAMN38169592 and SAMN38169593 for M4018 and R6-500, respectively.

**Supplementary information 2:**

**Comparative analysis of the biosynthetic gene clusters in *Streptomyces rimosus* M4018, R6 and parent strain ATCC 10970**

The locations of 46 putative BGCs are designated on the chromosome of three *S. rimosus* strains according to Slemc et. al. (2022) [21] (Supplementary Table 1). Partial deletion and chromosome rearrangements of the ends of the chromosomes of M4018 and R6-500 strains are displayed on Supplementary Fig. 1. A large chromosome deletion encompassing BGCs 1 - 8 is clearly visible in the M4018 strain. In addition to chromosome rearrangements in the strain R6-500, partial duplication of the end of the chromosome occurred, encompassing BGCs 1, 5, 6 and 7. Duplicated regions are located on the opposite extremities of the linear chromosome in R6-500. To identify and analyse putative BGCs of the industrial strains R6-500 and M4018, we analysed the genome assemblies using AntiMSASH 6.0 [27]. All BGCs identified in R6-500 and M4018 genomes were compared to ATCC 10970 strain according to Slemc et al. (2022) [21] (Supplementary Table 1), where a high-quality assembly of the ATCC 10970 genome, as well as comparison of giant linear plasmids from the three *S. rimosus* strains ATCC 10970, M4018 and R6500 were performed. There are 48 putative BGCs in the genome of S. *rimosus* ATCC 10970 [21], two of which are located on the large linear plasmid. According to our analysis, we identified 42 putative BGCs in the genome of M4018 and 2 BGCs are located on the linear plasmid. A large deletion in the right chromosomal arm was observed in the genome of the M4018 strain, resulting in loss of BGCs 5 - 8, encoding NRPS, PKS type lantipeptide and rimocidin, respectively (see Supplementary Table 1). Due to this deletion in M4018, BGC 9 encoding the oxytetracycline BGC (OTC) is located almost at the end of right chromosomal arm (Supplementary Fig. 1). Interestingly, BGCs 1-4 were relocated to the opposite chromosomal arm in an inverted orientation. In the new assembly of R6-500 we identified only 38 putative BGCs, some of which had relocated to different chromosome locations compared to ATCC 10970 or M4018 strains (Supplementary Fig. 1). Eight clusters are deleted in R6-500: BGCs 11-13 and 40-46, encoding lantipeptide, butyrolactone, PKS type I, PKS type I–NRPS, five NRPSs and a nucleoside cluster (see Supplementary Table 1). On the other hand, a DNA fragment containing BGCs 1-7 underwent DNA amplification. Due to the generation of a large inverted repeat at the end of the opposite genomic arm of R6-500, this genome now harbours two copies of the DNA fragment conferring BGCs 1-7 (Supplementary Fig. 1). Interestingly, despite the duplication events that took place in close vicinity to BGC 9 encoding the OTC biosynthesis and knowing that R6-500 was extensively screened for improved OTC titer, the OTC BGC is not duplicated, but rather relocated to the opposite chromosomal arm (Supplementary Fig. 1). In addition, we have confirmed that R6-500 lacks one of the plasmid-located clusters, BGC 2P [21], encoding an unknown metabolite.

**Supplementary information 3:**

**Deletion of 145kb region in *S. rimosus* ATCC 10970 by applying CRISPR-Cas9**

Both CRISPR constructs carried 2 consecutive gRNA cassettes, targeting both the 5′ and 3′ ends of deleted regions, and near-by homology regions with size of approximately 2000bp (Supplementary Table 3). To ensure better comparison between two engineered strains conferring 145kb and 240kb deletions, a gRNA cassette targeting the downstream region (oriented towards the core genome) and downstream homology region (DOWN) were identical for both plasmids/deletions, while the gRNA cassettes targeting upstream regions and the upper homology regions (oriented towards the left chromosomal arm) are specific for each plasmid/deletion. Exact coordinates of homology regions and gRNA sequences used are presented in Supplementary Table 3. By conjugation we introduced both pREP-CRISPR plasmids to *S. rimosus* ATCC 10970. The overall procedure for creating the deletions in *S. rimosus* using CRISPR-Cas9 was optimised before with the use of the Cas9-SD-GusA tool [26]. No additional induction of the theophylline inducible P1 promoter was necessary to achieve sufficient Cas9 expression in our system. Therefore, viable exconjugants could be characterised as mutants carrying 145kb/240kb deletions immediately after conjugation. Exconjugants were first cleared of pREP-CRISPR plasmids by sub-cultivation and only then assayed for the presence of the required deletion. In this way, we limited the exposure time of the *S. rimosus* genome to the active Cas9-gRNA complex, hence reducing the occurrence of non-specific (‘off target’) mutations and strain adaptation.

**Deletion of 145kb region in *S. rimosus* ATCC 10970 ∆OTC strain by applying CRISPR-Cas9**

To gain a deeper understanding of the profound effect of the 145kb deletion at the left sub-telomeric region of *S. rimosus* chromosome on OTC production, we introduced an identical 145kb deletion in the genome of the *S. rimosus* ATCC 10970 ∆OTC strain [28]. This is ATCC 10970 parent strain, which then had almost the entire *otc* BGC deleted and therefore does not produce oxytetracycline. After introduction of the pRep_P1_cas9_Δ145kb plasmid and sub-cultivation was completed, four independent clones of ATCC 10970 ∆OTC ∆145kb were verified with PCR (Supplementary Fig. 3). Morphologically stable strains were selected and finally three ATCC 10970 ∆OTC ∆145kb mutants (∆OTC_∆145kb_1, ∆OTC_∆145kb_2, ∆OTC_∆145kb_3) were further used in the *otc* BGC cluster re-introduction experiments.

**Supplementary information 4:**

**Confirmation of the anticipated deletion events by PCR and DNA sequencing of the PCR products.**

PCR reactions were performed on isolated genomic DNA samples. Importantly, the corresponding “Fw” primer in all PCR conformations was designed to anneal outside of the upper homology regions to ensure reliable results (see Supplementary Fig. 3). 3 independent isolates of ATCC 10970 and 4 independent isolates of ATCC ΔOTC strain displayed the anticipated Δ145kb genotype, indicated by an amplicon of 2168 bp (Supplementary Fig. 3). In the case of the 240kb deletion strain, 6 clones isolated displayed the anticpated genotype, with PCR amplicon of 2334 bp. As expected, in the case of the ATCC 10970 parent strain (negative control), only non-specific amplicons were observed (Supplementary Fig. 3). To ensure PCR amplified DNA fragments were undoubtedly correct, especially due to occurrence of non-specific amplicons, PCR amplicons of the predicted sizes (Supplementary Fig. 3) were excised from the gel, isolated, and analysed by Sanger sequencing (Macrogen, Inc.; Daejeon, Republic of Korea). Without exception, the predicted DNA sequences, corresponding to precise deletion of the 145kb and 240kb regions were present in all sequenced amplicons. Finally, as described in Supplementary information 5, by applying whole genome sequencing and/or whole genome transcription analysis, we have confirmed that deletions of 145kb and 240kb were indeed introduced correctly into the ATCC 10970 parent strain. To verify the anticipated genome reduction, 10 plasmid-cleared independent exconjugants were analysed for each deletion size. For gDNA extraction an agar plug of sporulating colonies from MS medium was inoculated into 5 mL TSB and harvested during mid-exponential-growth phase (after 24 h). gDNA was isolated from potential mutants using a peqGOLD bacterial DNA isolation kit (VWR, USA). The presence of the expected deletion was confirmed on isolated gDNA samples by PCR using RepliQ HIFI Though mix (Quantabio, USA). In the case of the 145kb deletion, primer pair Δ145kb_UP_Fw and Δ145kb_DOWN_Rw was used to identify mutants with anticipated deletion size. Similarly, primer pairs were used to PCR-amplify regions that underwent the planned recombination after 240 kb deletion and *rimA* deletion experiments. The primer sequences used for confirmation of all deletions and expected amplicons sizes are represented in Supplementary Table 4 and 10. PCR amplicons of the predicted size were excised from agarose gels, isolated using E.Z.N.A. Gel Extraction Kit (Omega Bio-Tek, USA) and analysed by Sanger sequencing at Macrogen, Inc. (Daejeon, Republic of Korea).

**Supplementary information 5:**

**Whole genome sequencing and analysis of 145kb deletion strains**

To analyse the genomic outcomes of CRISPR-Cas9 editing in the *S. rimosus* ATCC 10970 Δ145kb strain, WGS was performed as described in the Methods section. gDNA isolated from Δ145kb mutants together with two ATCC 10970 controls was sequenced using the Illumina platform with up to 200x average coverage, to enable in depth analysis of possible mutations. Illumina sequencing generated close to 14 million short reads per sample/strain, at least 94.28% of which were of high quality (Q20) and at least 87.76% of which were of very high quality (Q30). Mapped assembly was obtained by aligning reads to the reference grade *S.* *rimosus* ATCC 10970 genome [21].

**Supplementary information 6:**

**Cultivation, sampling and isolation of mRNA**

To understand better the differences between ATCC 10970 and strains with 145kb and 240kb deletions, we carried out comparative transcriptional analysis. To ensure adequate amounts of sample cultures, all strains were cultivated in 250mL Erlenmeyer flasks and in three replicates. The ATCC Δ145kb strain performed even better under shake flask conditions and on average produced 3.56 g/L OTC after 124h (5days), whereas the OTC titers of ATCC Δ240kb (0.62 g/L) and parent ATCC 10970 (0.2 g/L) strains were lower in 250mL shake flasks compared to fermentations in 50mL tubes. To establish the optimal sampling times, we measured titers of OTC during the entire production stage (from 20h to 144h of incubation time) (Supplementary Fig. 7). Compared to the ATCC 10970 strain, we observed a remarkable early induction of OTC biosynthesis by the ATCC Δ145kb strain, which already reached a titre of around 1g/L OTC at 44 hours of incubation. We therefore confirmed the ATCC Δ145kb strain initiates OTC biosynthesis earlier than the parental ATCC 10970, which is a common property of industrial antibiotic high-producing strains. Based on OTC production curves (Supplementary Fig. 7) we selected two time points for sampling and RNA isolation: 24 hours and 50 hours of the fermentation. The 24-hour time point was selected as the early exponential time point (see Supplementary Fig. 5A) and was set to capture OTC-related gene expression in the highest performing mutant, ATCC Δ145kb. It is important to note, that at 24 hours of incubation, little OTC was detected in the culture of the parent strain ATCC 10970 (Supplementary Fig. 7). However, at 50 hours of incubation, we aimed to capture gene expression in late exponential phase, when the ATCC 10970 strain starts to produce quantifiable amounts of OTC. Cultures were fixed immediately after sampling. Total RNA was isolated, evaluated for quality and submitted to Novogene (China) for library preparation and sequencing. Total RNA was isolated with a RNeasy Mini Kit (Qiagen), as described in Supplementary information 6 from 0.8 ml of the fixed culture. Before isolation, the cell pellet was washed in fixative and then treated with lysozyme (40 mg/mL, Sigma) and proteinase K (20 mg/mL; Macherey-Nagel) for 10 minutes, followed by homogenization with FastPrep using Lysing Matrix B beads (MP Biomedicals) and RLT Buffer (750 μL, Qiagen). The homogenate was centrifuged, and the supernatant was used for subsequent RNA isolation using the RNeasy Mini Kit (Qiagen) with the following modifications to the manufacturer’s protocol: samples were washed twice and three times with RW1 and RPE buffer, respectively. RNA was eluted after 5 min incubation with pre-warmed (65°C) RNAse-free water. Samples were further purified with DNase I and the Zymo RNA Clean & Concentrator-5 kit (Zymo Research), with DNase treatment in solution and washing repeated three times. The efficiency of DNase digestion was tested using RT- controls. Quantity and quality of RNA were tested using Nanodrop, gel electrophoresis and Bioanalyzer (Agilent).

**Supplementary information 7:**

**Gene ontology (GO) analysis of RNA-seq data:**

GOs were annotated using Blast2Go to each down/upregulated gene with log^2^ fold ratio >2/ <-2 in the first sampling time point (24h). The list of GO-terms was then submitted to the REVIGO tool (<http://revigo.irb.hr/>) for summarisation of GO-terms and determination of affected biological functions. For the Δ145kb strain, GO-terms were used that occurred 2 or more times. For strain Δ240kb, where GO terms were not abundant, each GO term was included in analysis. The results are presented as Supplementary Data 1.

Clusters obtained by hierarchical clustering (see Fig. 5) consisting of more than 10 genes were analysed using STRING (https://version-11-5.string-db.org), on the uploaded *S. rimosus* ATCC 10970 proteome (available under STRING proteome Id: STRG0044NQX), but no relevant results were obtained by this type of analysis.

**Supplementary information 8 – Methods:**

**Diphenylamine-colorimetric method for DNA content assay**

The procedure for DNA quantification from samples was based on Burton (1956) [75]: 1 mL of the culture broth was centrifuged in 2 mL tubes at 16,000 x g for 10 minutes to pellet the cellular components, while the supernatant was removed. The pellet was washed twice with 1 mL of distilled water, followed by centrifugation at 16,000 x g for 10 minutes after each wash. After the washing steps, the pellets were resuspended in 2 mL of 0.9% NaCl to obtain a uniform cell suspension. A 10x dilution was achieved by mixing 100 μL of the cell suspension with 900 μL of 0.9% NaCl. 400 μL of both the diluted and undiluted cell suspensions were transferred to separate 2 mL tubes. To each tube, 400 μL of HClO_4_ was added, and the contents were mixed thoroughly. Incubation of the tubes was carried out at 70 °C for 20 minutes to facilitate cell lysis and DNA release. Subsequently, 800 μL of freshly prepared diphenylamine reagent was added to each tube, and the tubes were inverted to ensure complete mixing of the reagent with the sample. The tubes were then incubated at 30 °C for 16 hours to allow the formation of the diphenylamine-DNA complex. Centrifugation at 16,000 x g for 5 minutes was performed, and the supernatant was carefully transferred to fresh tubes. 200 μL of the supernatant from each tube was transferred to a microplate for absorbance measurement at 600 nm using a microplate reader (Tecan Spark, Switzerland).

**Re-introduction of the *otc* gene cluster in the *S. rimosus* ATCC10970 Δotc Δ145kb and *S. rimosus* ATCC Δ145kb strains**

In previous work described by Pikl et al., (2021) [28] the entire *otc* cluster was captured from *S. rimosus* genomic DNA and assembled into the pYAC-ΦC31-Ts-OTC vector using a TAR (transformation assisted recombination) approach. Here, the pYAC-ΦC31-Ts-OTC plasmid was introduced to 3 independent isolates of *S. rimosus* ATCC 10970 ΔOTC Δ145kb and *S. rimosus* ATCC Δ145kb. The plasmid was first introduced to *E. coli* ET12567-pUB307, that mediated the conjugal transfer to recipient *S. rimosus* spores. Exconjugants were patched and selected on MS +thiostrepton (30 ug/mL) +nalidixic (30 ug/mL) agar plates and were in the following stages cultivated on MS medium with addition of 30 ug/mL thiostrepton. During OTC fermentation of these strains, a lower amount (5 ug/mL) of thiostrepton was added only to vegetative GOTC-V medium. Three independent *S. rimosus* ATCC 10970 ∆OTC strains with a precisely deleted *otc* gene cluster and containing the 145kb deletion were complemented with th epYAC-ΦC31-Ts-OTC vector containing the entire OTC BGC at the ΦC31 *attB* site. Altogether, the ΦC31-mediated integration of pYAC-ΦC31-Ts-OTC in ATCC 10970 ∆OTC ∆145kb engineered strains resulted in re-location of the *otc* BGC from left terminal arm to *attB* site-located in the central region of the *S. rimosus* genome [28]. Since the entire genome contains only one perfect *attB* site, the location of pYAC-ΦC31-Ts-OTC was not additionally verified in our study. After introduction of the pYAC-ΦC31-Ts-OTC plasmid into the ATCC 10970 ∆OTC ∆145kb strain, it re-gained a brown pigmentation, which is characteristic of the OTC production colony phenotype. Duplication of the *otc* BGC was achieved by introducing plasmid pYAC-ΦC31-Ts-OTC plasmid into the ATCC ∆145kb strain.

**Supplementary Figure 1**

***S. rimosus* M4018 and R6-500 assemblies plotted against *S. rimosus* ATCC 10970**


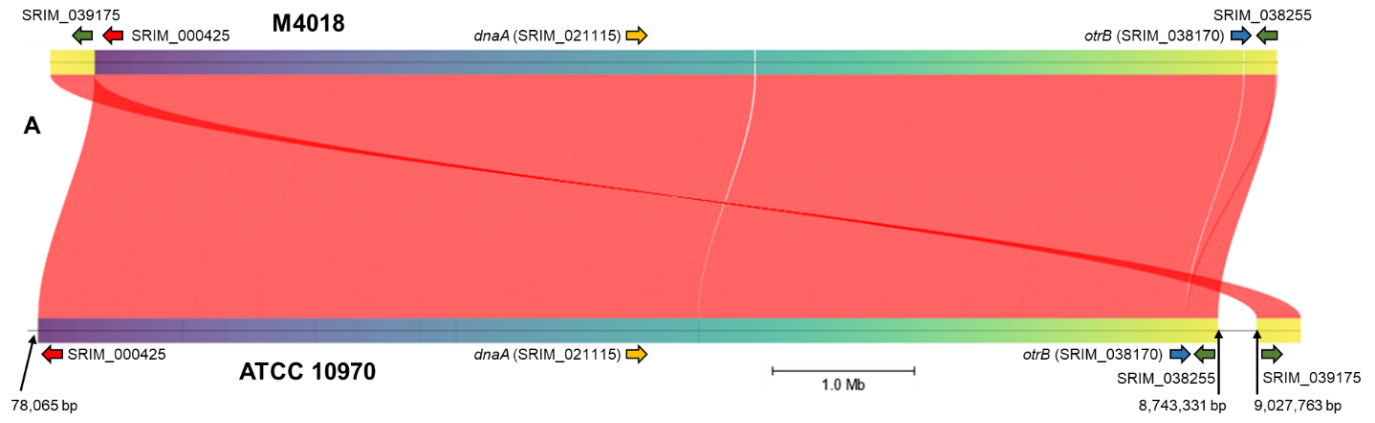


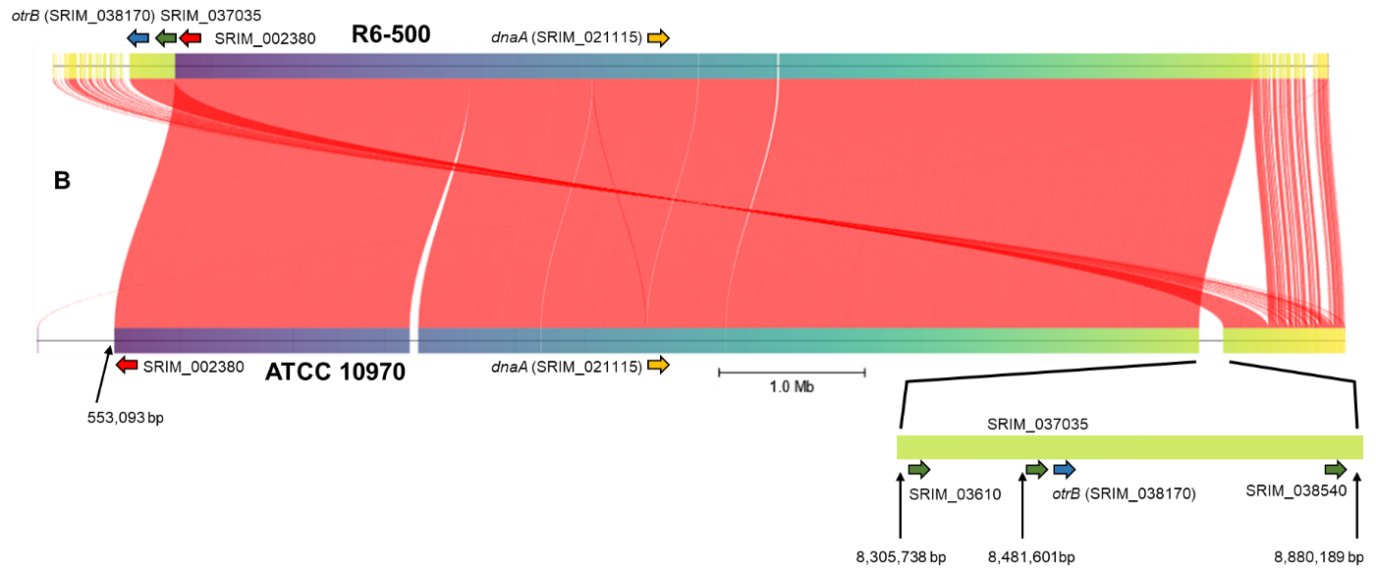


***S. rimosus* M4018 (A) and R6-500 (B) assemblies plotted against *S. rimosus* ATCC 10970 reveal extensive rearrangements at the chromosome ends.**  Both M4018 and R6-500 assemblies were compared with ATCC 10970 using using FastANI [76] hosted within Proksee (<https://proksee.ca/>). All genomic locations and gene identifiers are from ATCC 10970. Genes found in the left and right chromosomal arms of this strain are displayed in red and green respectively, whilst *dnaA* indicating *oriC* and *otrB* (that indicates the OTC cluster) are displayed in orange and blue, respectively. *otrB* is a resistance gene which acts by efflux of the OTC, located at one end of the OTC cluster [77]. The region from 8,305,738 bp and 8,880,189 bp (ATCC 10970 locations) is zoomed in for M408 to allow a better display of this region.

**Supplementary Figure 2**

**AntiSMASH analysis of relevant un-known BGCs**


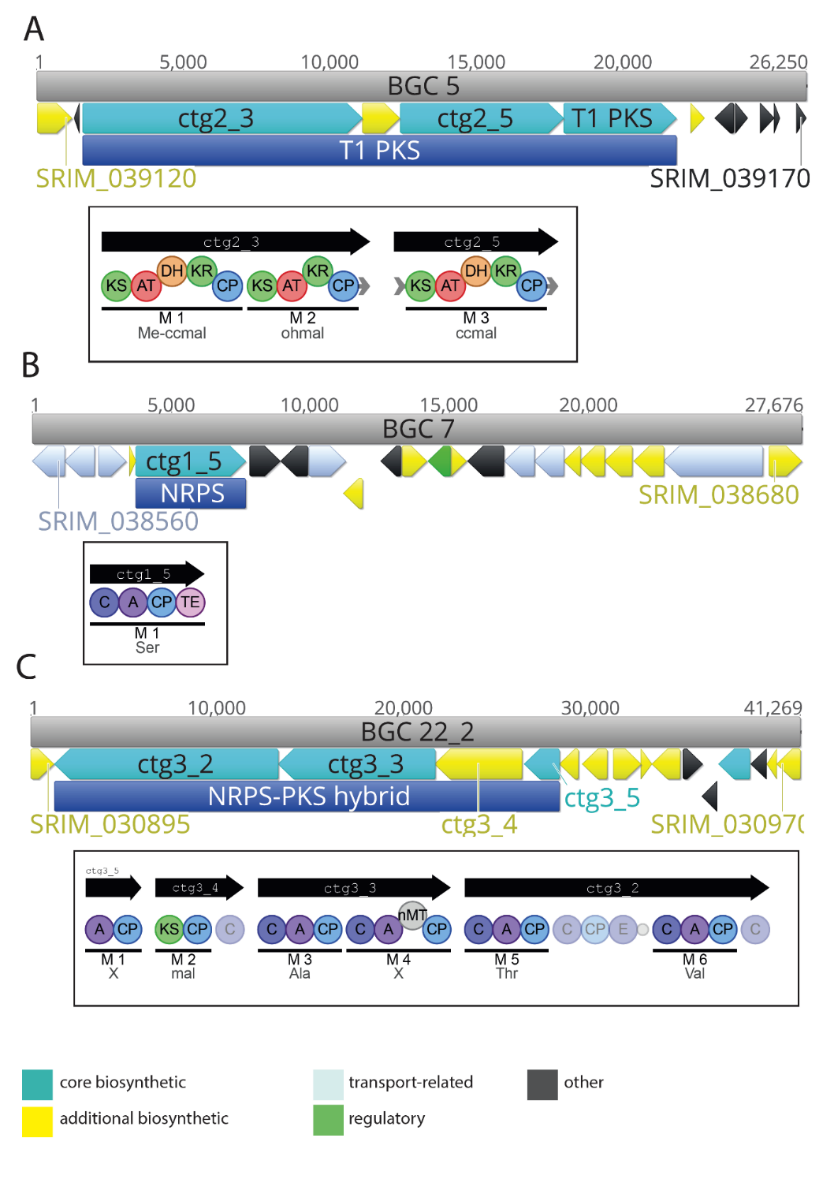


AntiSMASH analysis of BGCs 5 (A), 7 (B) and 22_2 (C) encoding unknown metabolites. BGCs A and B were removed as part of the 145kb and 240kb deletions. BGC 22_2 is alongside longicatenamycin B/C cluster the most overexpressed BGC in engineered strains (see also **Supplementary Fig 6.1**).

**Supplementary Figure 2.1**

Visualisation of overexpression of entire BGC22 in ATCC Δ145kb strain


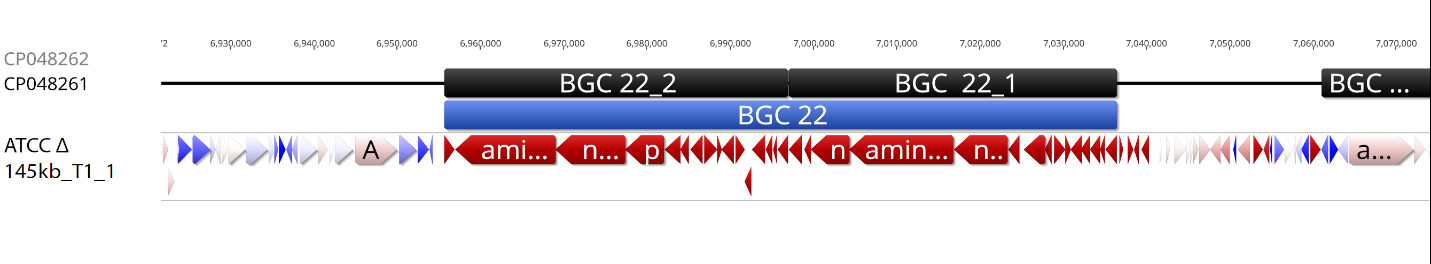


Visualisation of overexpression of entire BGC22 in the ATCC Δ145kb strain (T1) with Geneious software (R11.1.5; <https://www.geneious.com>). Red annotations-significant overexpression, white annotations – neutral, blue annotations – down-regulation.

**Supplementary Figure 3**

**PCR verification of performed large deletions and *rimA* inactivation**


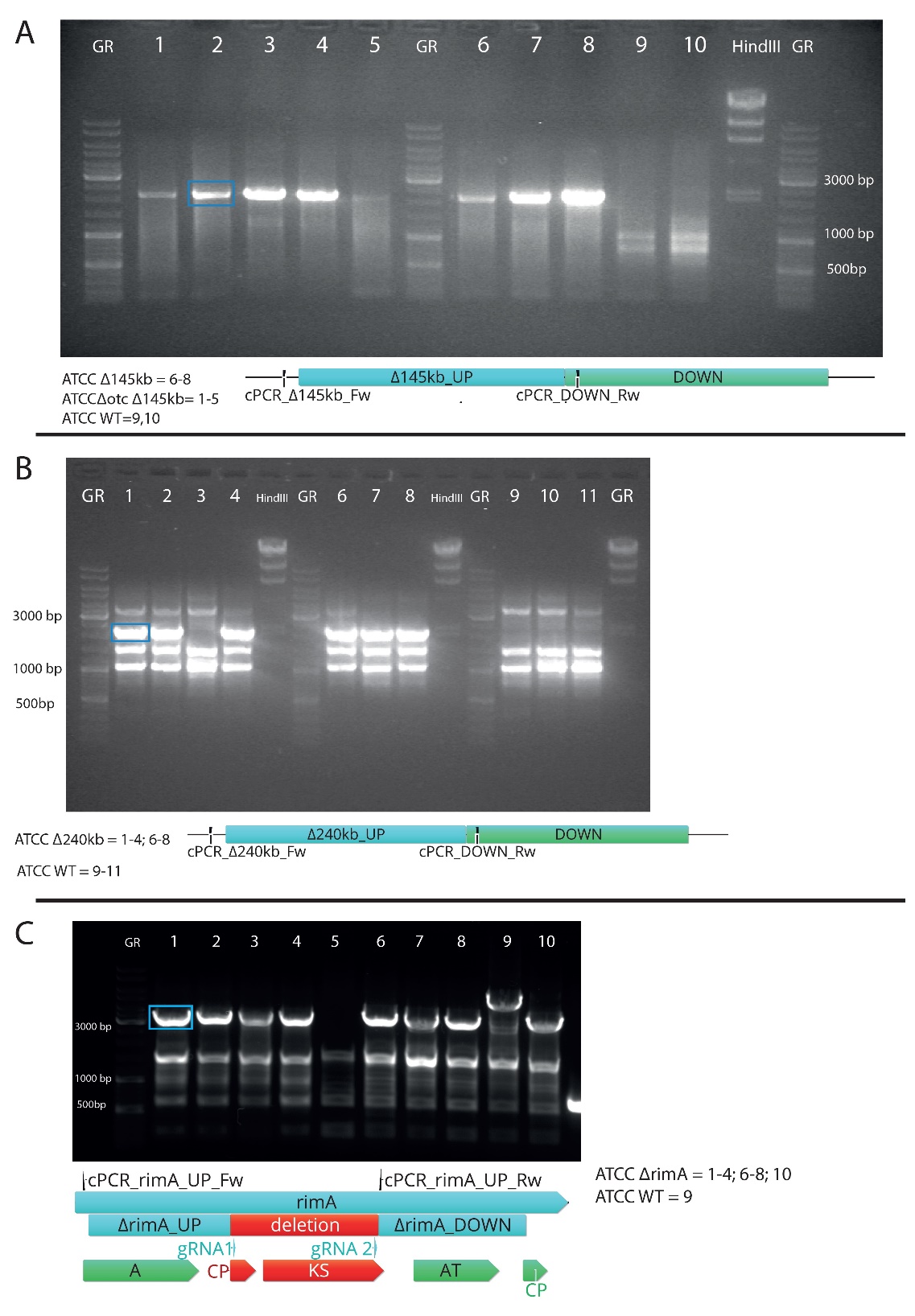


PCR verification of *Δ145kb (A), Δ240kb* (B) and Δ*rimA* (C) genotype in *S. rimosus* transformants after the CRISPR-editing procedure. Schematic representations of each targeted region are presented with corresponding primer pairs. The predicted size of the PCR amplicons, numbers of tested and verified mutants for 145kb, 240kb and *rimA* deletions are presented in Supplementary Table 4. Primer sequences are listed in Supplementary Table 10. Blue squares denote the correct PCR amplifications for the edited genotype.

**Supplementary Figure 4**

**OTC production titer and transcriptomics results of *Streptomyces rimosus* ATCC Δ145kb-b.**

**Note*** Results in main text are presented only for best performing strain - ATCC Δ145kb, while OTC production and transcriptomics was also performed for another independent strain with 145kb deletion, designated as ATCC Δ145kb-b.


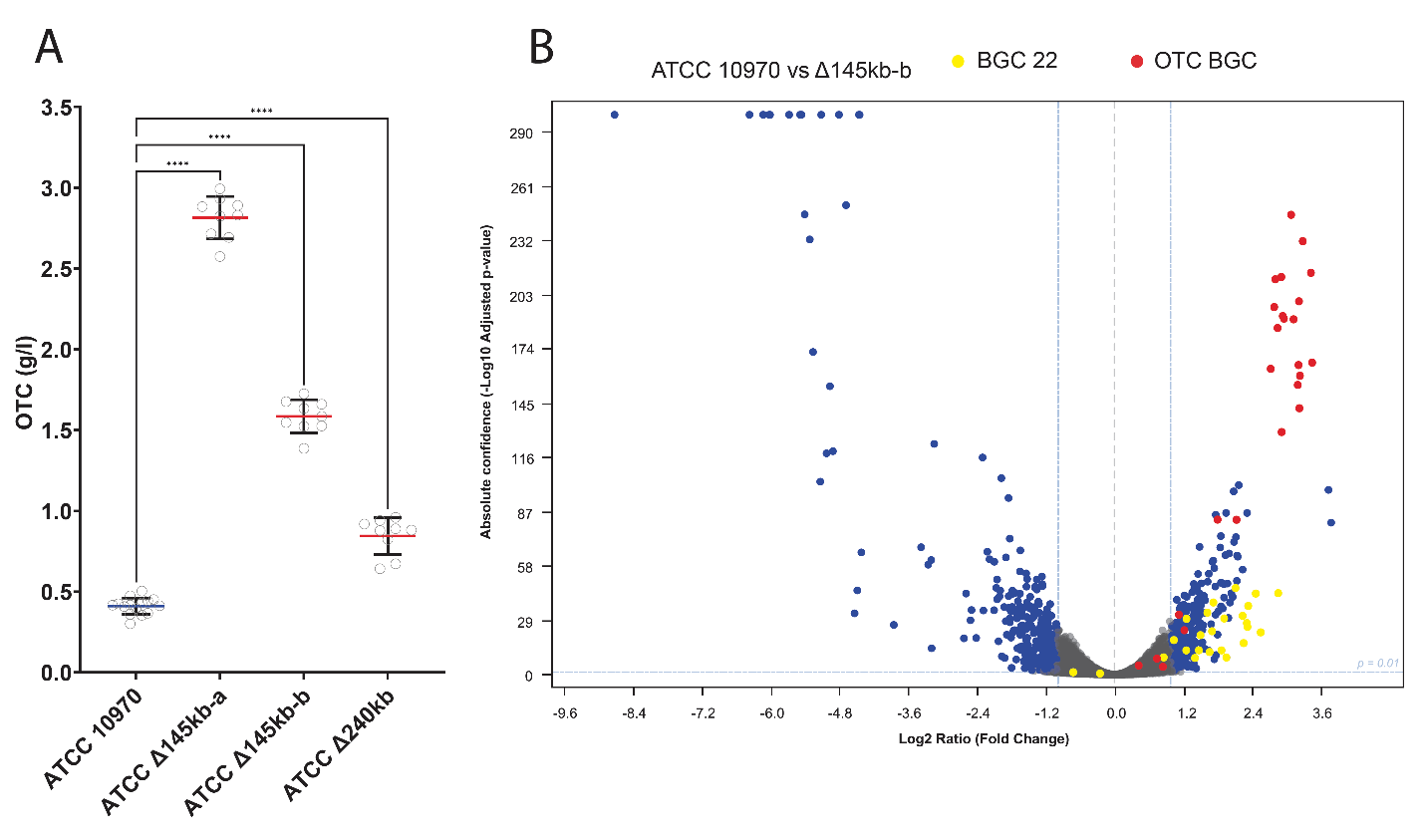


**A** OTC yield from fermentation experiment including ATCC Δ145kb-b strain. **B** Volcano Plot depicting transcriptome changes when comparing ATCC Δ145kb-b to control ATCC 10970. The x-axis represents the log2 fold change in gene expression, the y-axis represents the statistical significance as -log 10 adjusted p-value. Each data point represents an individual gene, and significant differentially expressed genes are highlighted (log2 ratio >1, log2 ratio <-1, log10 p value > 2). Strong overexpression of BGC 22 and OTC BGC is consistent with those presented for ATCC Δ145kb (-a) strain in the main text, thus indicating relevance of 145 kb deletion.

**Supplementary Figure 5**

**Comparison of growth characteristics of ATCC 10970 and engineered strains in OTC fermentation**


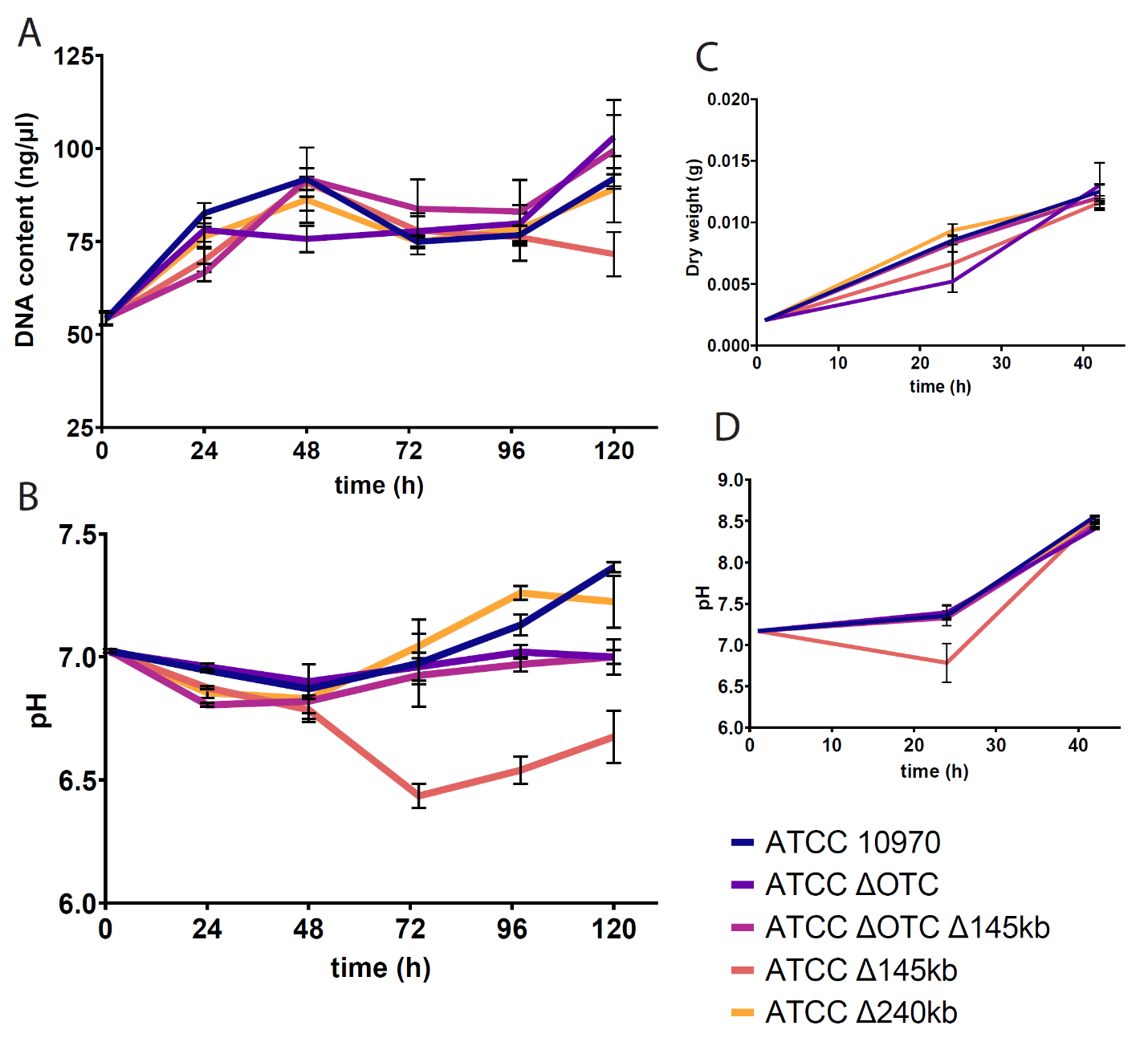


Comparison of growth characteristics of ATCC 10970 and engineered strains with 145kb, 240kb and *otc* deletion during OTC fermentation. A) DNA content (diphenylamine-colorimetric method) of samples from engineered and control strains during production stage of OTC fermentation. B) pH curves of strains during production stage of OTC fermentation. C) Dry biomass of strains during vegetative stage of OTC fermentation. D) pH curve of strains during vegetative stage of OTC fermentation. Measurments were performed from two biological replicates.

**Supplementary Figure 6**

**Morphology of ATCC 10970 and engineered strains**


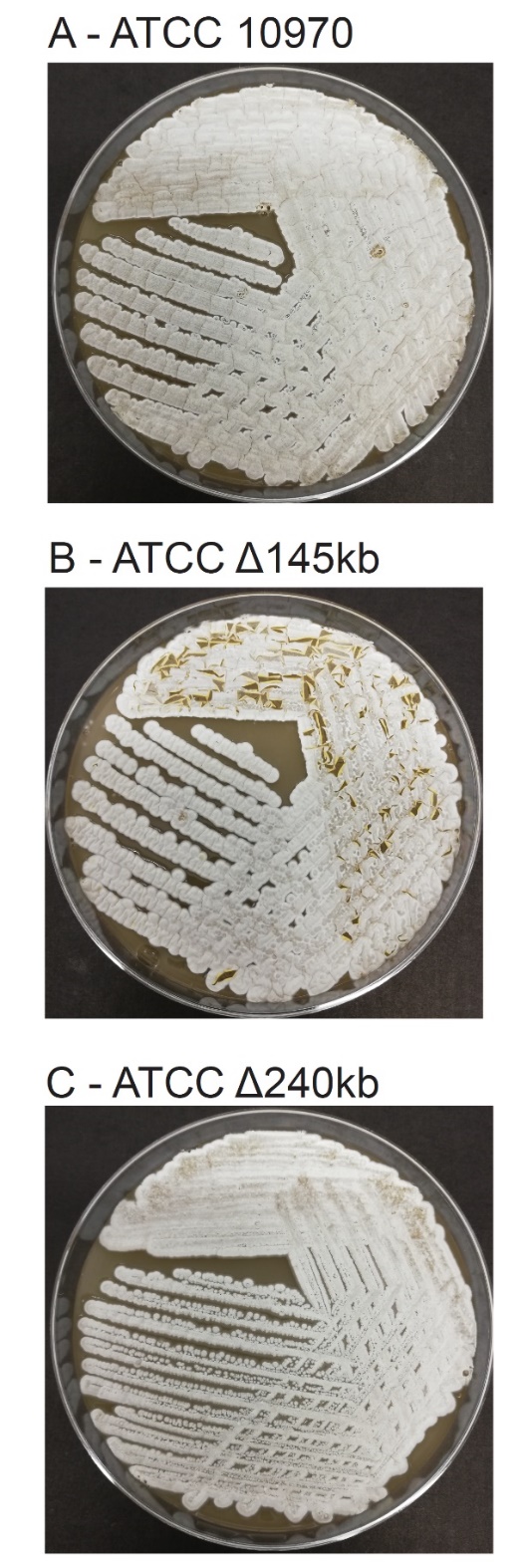


Morphology of engineered strains ATCC Δ145kb (**B**), ATCC Δ240kb (**C**) and *S. rimosus* ATCC 10970 (**A**) on SM agar plates after 7-day incubation. Notable differences can be observed between ATCC Δ145kb (**B)** compared to WT (A) or ATCC Δ240kb (**C**).

**Supplementary Figure 7**

**Measurements of OTC concentrations during fermentation of ATCC 10970 and engineered strains**


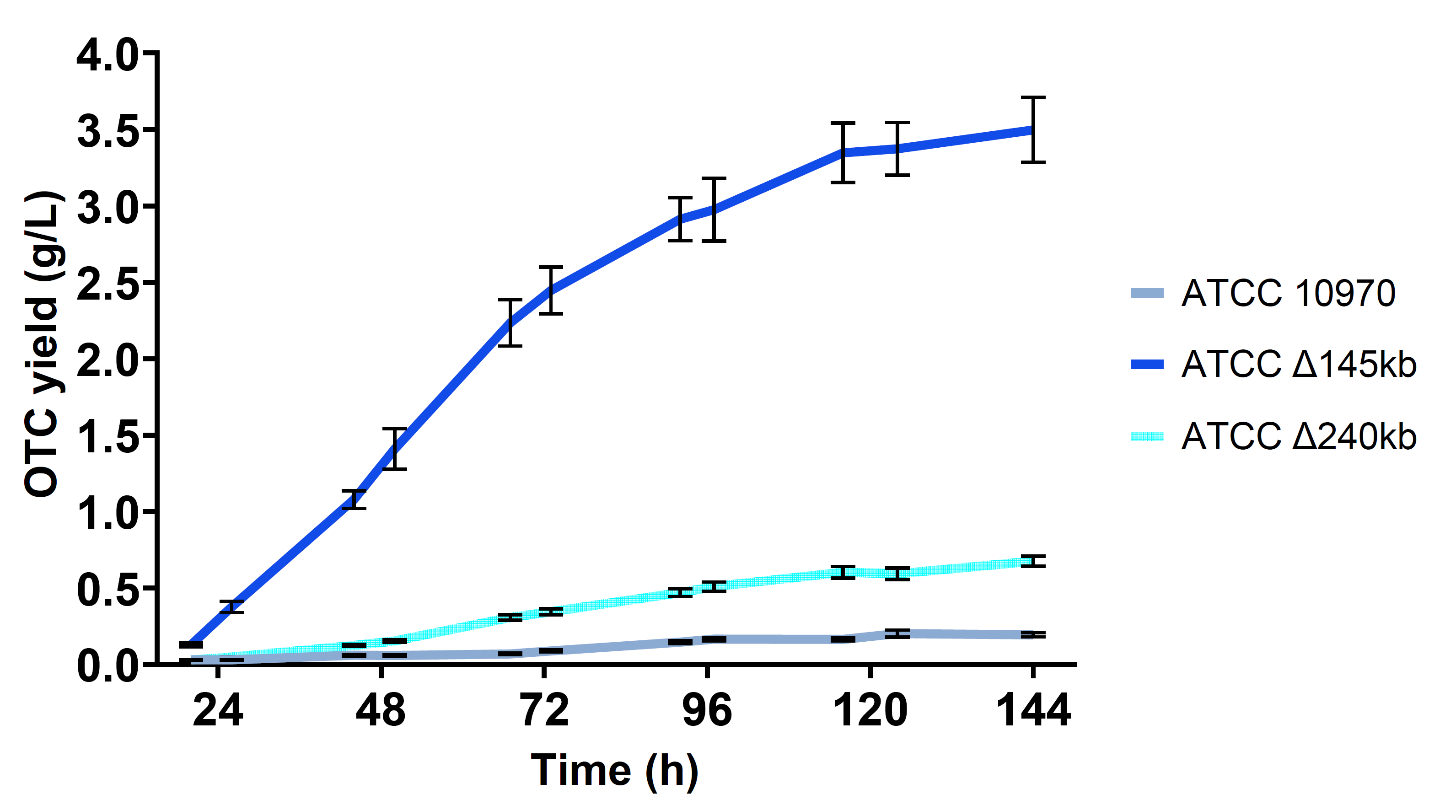


Comparison of OTC titer during the entire production stage (from 20h to 144h of incubation time) between engineered strains and ATCC 10970. Measurments were performed from two biological replicates.

**Supplementary Figure 8**

**Principal Component Analysis (PCA) plots from transcriptome analysis**


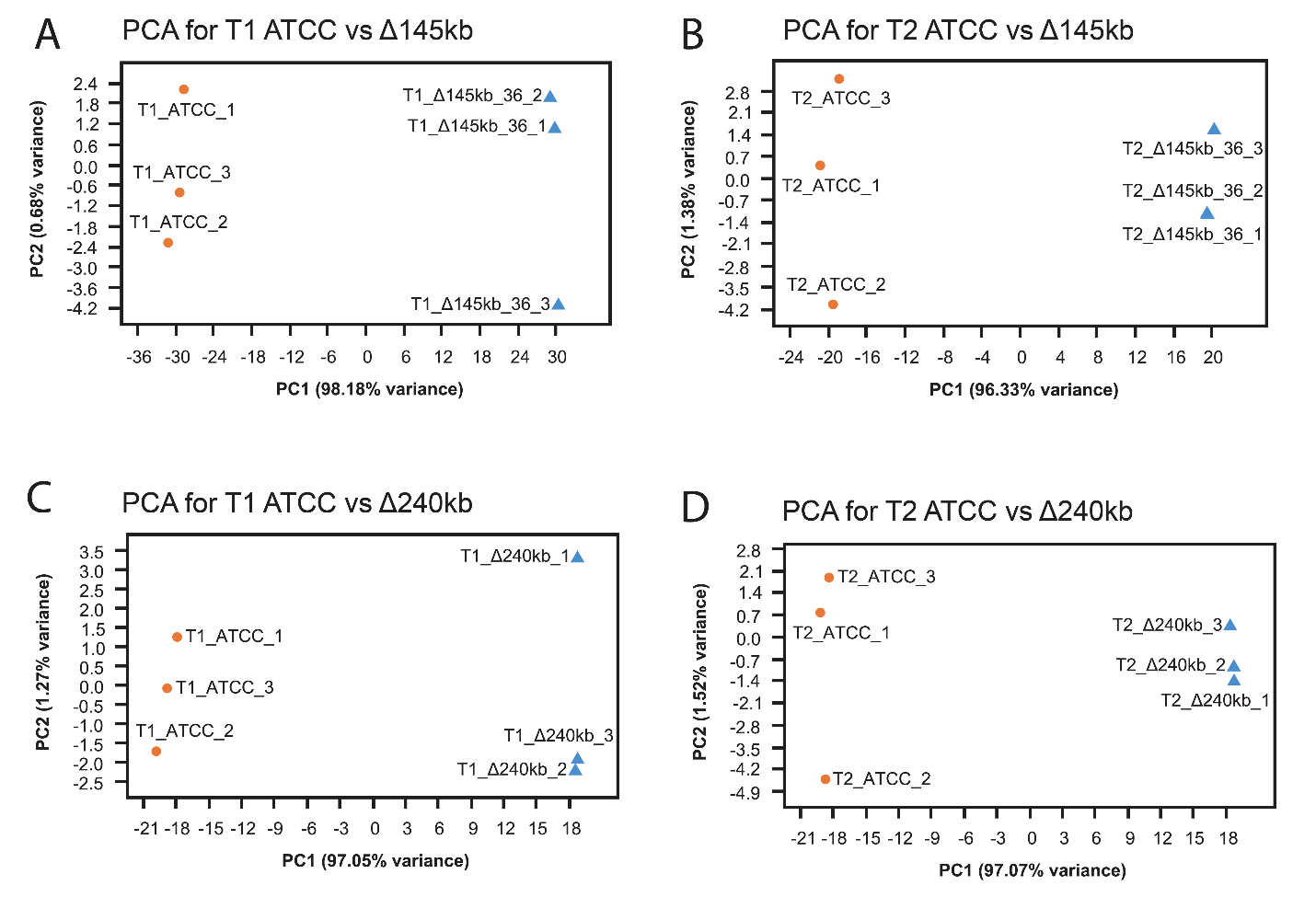


Principal Component Analysis (PCA) plots, displaying most significant patterns of variation in the original RNA-seq data between samples.

**Supplementary Figure 9**

**Comparison of DNA content in OTC production broths from ATCC 10970 and engineered strains in the transcriptomics experiment**


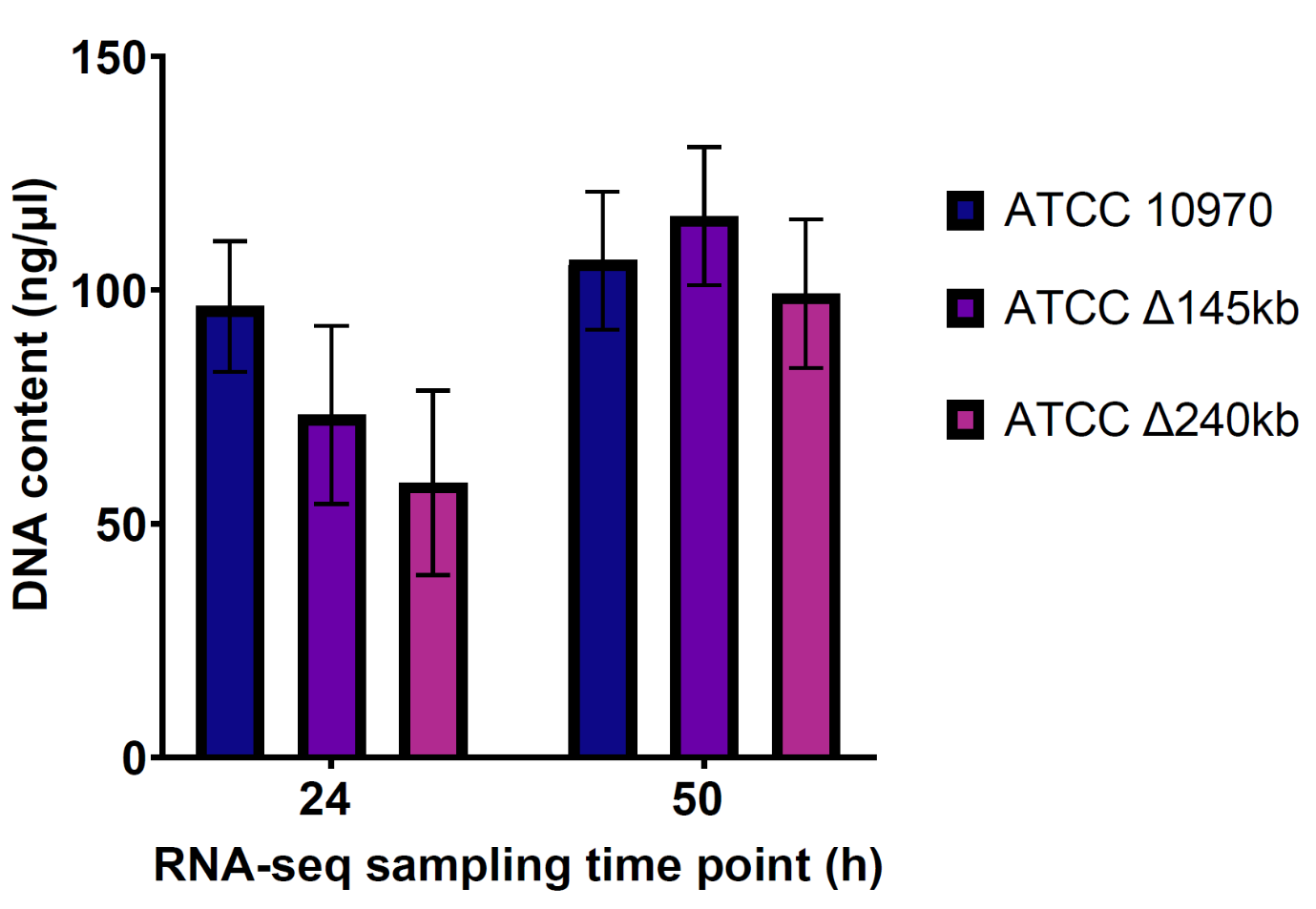


Comparison DNA content (diphenylamine-colorimetric method) directly from cultures engineered and control strains used for transcriptome analysis at first (24h) and second (50h) time point. Measurments were performed from two biological replicates.

**Supplementary Figure 10**

**Number of differentially expressed genes (DEGs) in 100kb interval across the genomes of engineered strains.**


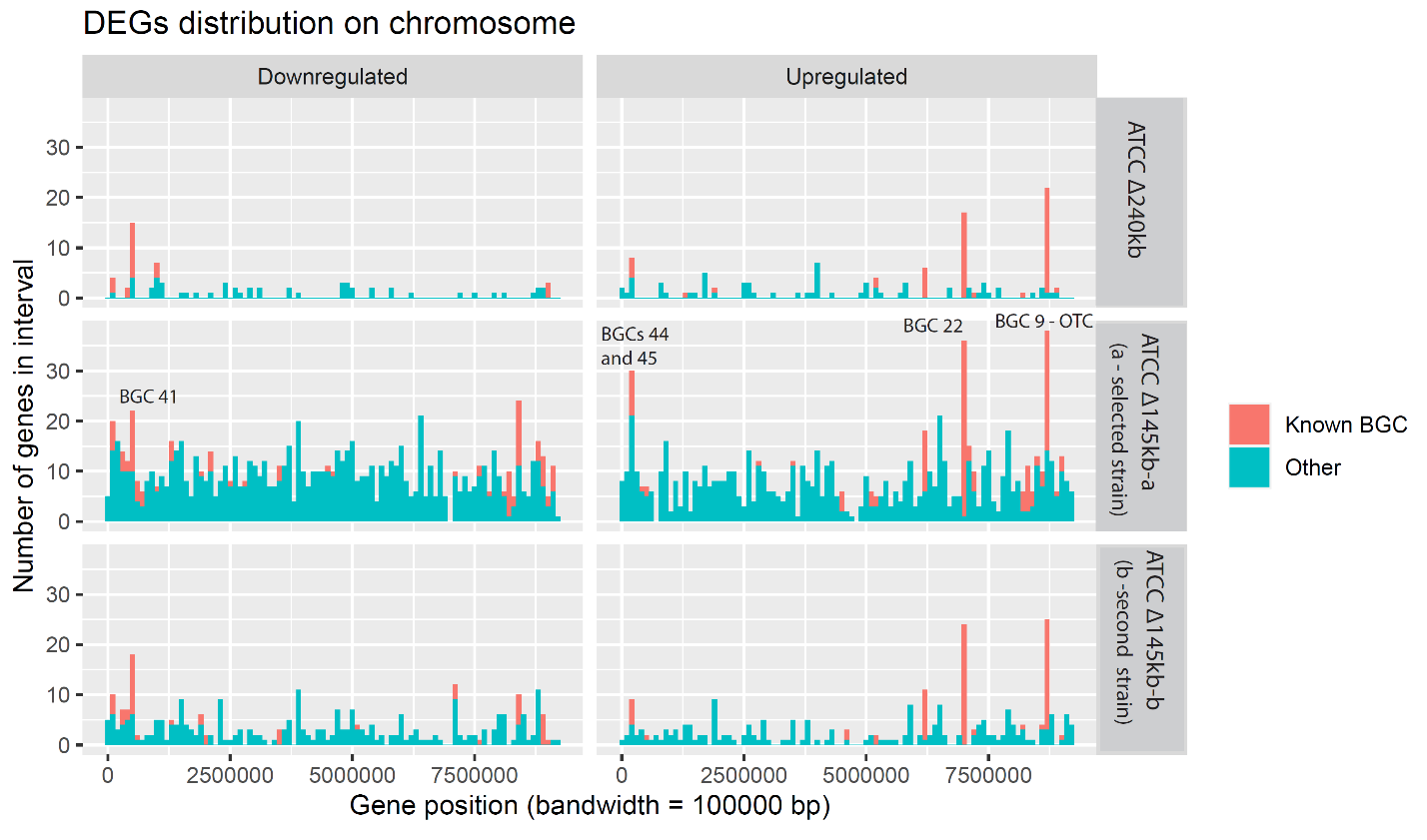


Number of differentially expressed genes (log^2^ values >1/ <-1 in at least one strain at T1) in an interval is plotted together with genome location (bandwidth = 100000bp). This type of analysis targets BGCs, which consist of large number of consecutive genes.

**Supplementary Figure 11**

**Chromatograms from full-scan LC-MS analysis of aqueous phase**


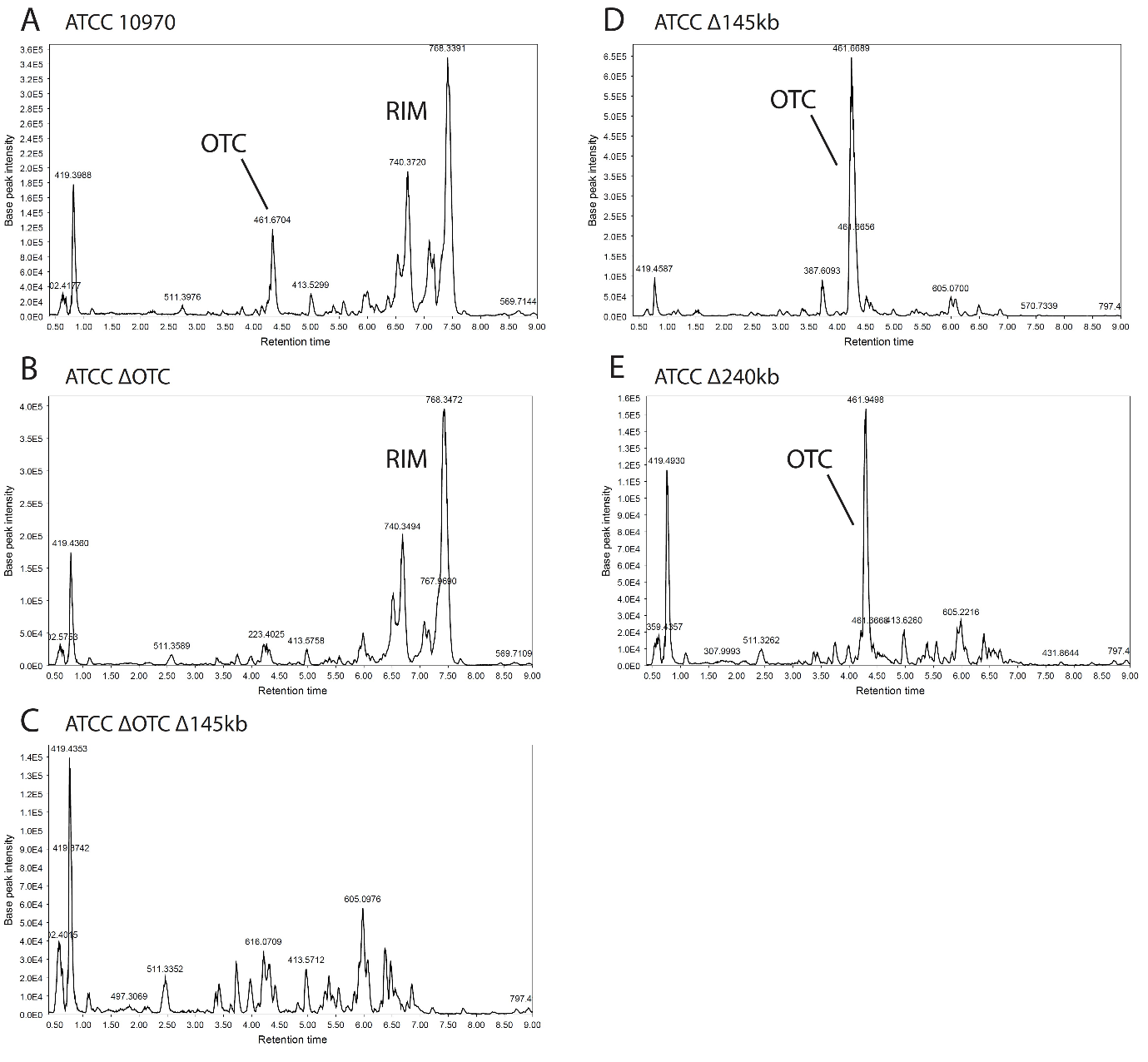


Chromatograms from full-scan LC-MS analysis OTC production broths of control and engineered *S. rimosus* strains: ATCC 10970 (A), ATCC ΔOTC (B), ATCC ΔOTC Δ145kb (C), ATCC Δ145kb (D), ATCC Δ240kb (E). **Culture supernatants were analysed.** OTC – oxytetracycline, RIM- rimocidins.

**Supplementary Figure 12**

**Chromatograms from full-scan LC-MS analysis after acetonitrile (ACN) extraction**


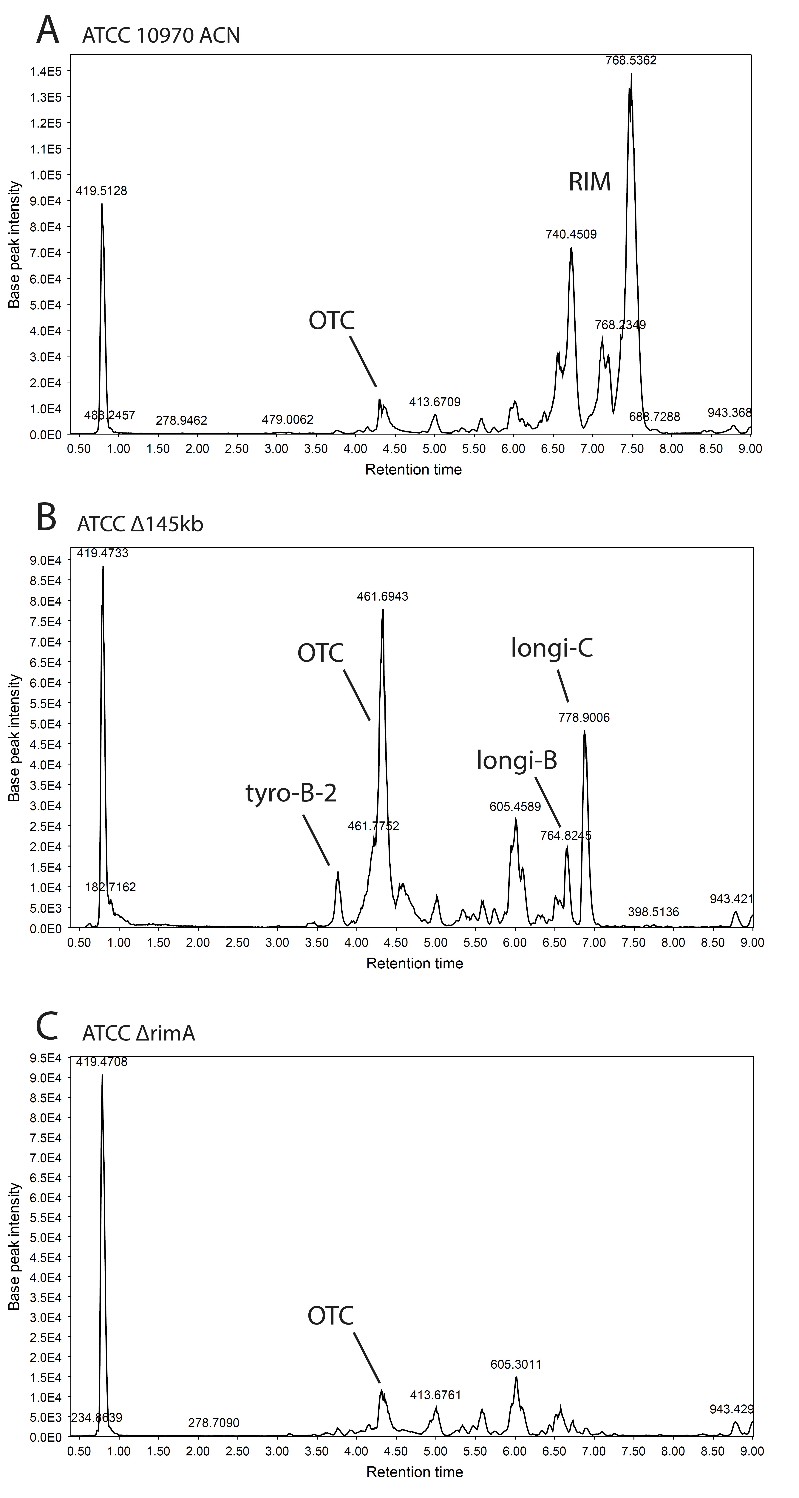


Chromatograms from full-scan LC-MS analysis OTC production broths of control and engineered *S. rimosus* strains: ATCC 10970 (A), ATCC Δ145kb (B), ATCC Δ*rimA* (C). **Acetonitrile extracts (4:1 vol) were** analysed. OTC – oxytetracycline, RIM- rimocidins, tyro-B-2 – tyrobetaine – 2, longi-B - longicatenimycin B, longi-C – longicatenamycin C.

**Supplementary Figure 13**

**Chemical structures of rimocidins (RIM) and pimaricin**


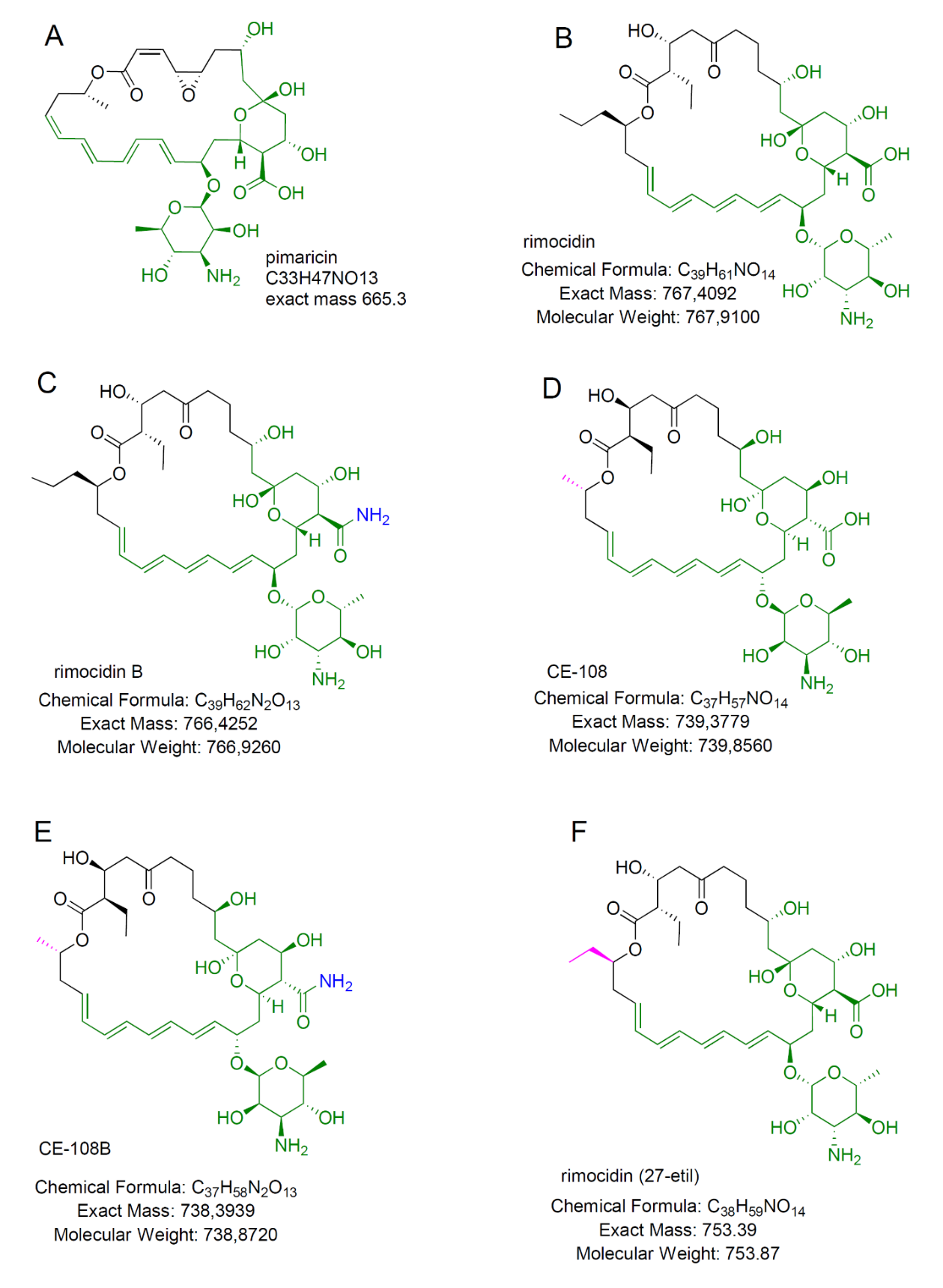


Chemical structures of rimocidins (B-F), detected in *S. rimosus* ATCC 10970 background (HRMS data in Supplementary Table 7). (A) pimaricin (natamycin) – possible standard for MS fragmentation analysis of rimocidins due to similar structure (marked green).

**Supplementary Figure 14**

**OTC yields after modification of GOTC-P medium.**

OTC yield improvement after modification of GOTC-P medium; Addition of 40 g/L Corn starch (CS) and 10-50 mg/L Amylase (Amy) improved titer of the best performing strain ATCC Δ145kb+φC31*otc*, but not OTC yields from type strain ATCC Δ145kb. Only a modest increase in OTC yield was observed for ATCC Δ145kb strain. These results indicate, that in our case, additional carbon source significantly improves OTC production only in strain with the highest expression of *otc* genes.

**Supplementary Table 1.** Biosynthetic gene clusters in *S*. *rimosus* ATCC 10970, M4018 and R6-500 chromosome and plasmid

| **No. in ATCC 10970** | **Absent in M4018** | **Absent in R6-500** | **Type** | **Start_ATCC locus tag (SRIM_)** | **End_ATCC locus tag (SRIM_)** | **Most similar known biosynthetic gene cluster (percent of similarity)** |
| --- | --- | --- | --- | --- | --- | --- |
| 1 |  |  | NRPS fragment | 040090 | 040110 | Paromomycin (7 %) |
| 2 |  |  | PKS type I-NRPS | 039615 | 039665 | NA |
| 3 |  |  | Terpene | 039570 | 039605 | Isorenieratene (85%) |
| 4 |  |  | NRPS | 039485 | 039515 | Atratumycin (13%) |
| 5 | X |  | Type I PKS | 039120 | 039170 | Sceliphrolactam (32 %) |
| 6 | X |  | Type I PKS | 038745 | 039060 | Rimocidins (100 %) |
| 7 | X |  | NRPS | 038560 | 038680 | Qinichelins (22 %) |
| 8 |  |  | Lassopeptide | 038340 | 038375 | Lagmysin (80 %) |
| 9 |  |  | Type II PKS | 038060 | 038175 | Oxytetracycline (100 %) |
| 10 |  |  | Type I PKS | 037340 | 037425 | NA |
| 11 |  | X | Lantipeptide | 036840 | 036870 | NA |
| 12 |  | X | Type I PKS | 036685 | 036780 | Spiroindimicins (6 %) |
| 13 |  | X | NRPS like | 036430 | 036495 | Stenothricin (13 %) |
| 14 |  |  | NRPS-PKS type | 036275 | 036340 | Rimosamide (92 %) |
| 15 |  |  | NRPS | 035930 | 036135 | Daptomycin (14 %) |
| 16 |  |  | arylpolyene | 035605 | 035855 | Herboxidiene (3 %) |
| 17 |  |  | Terpene | 034850 | 034895 | Hopene (76 %) |
| 18 |  |  | NRPS | 034025 | 034100 | Isocomplestatin (93 %) |
| 19 |  |  | Melanin | 033360 | 033395 | Bagremycin A/B (11%) |
| 20 |  |  | Lantipeptide | 031530 | 031570 | NA |
| 21 |  |  | NRPS | 031170 | 031225 | Streptobactin (70 %) |
| 22 |  |  | NRPS | 030895 | 031065 | Longicatenamycin B/C + NA |
| 23 |  |  | NRPS-PKS type | 027955 | 028010 | Tyrobetaine (100 %) |
| 24 |  |  | NRPS | 023120 | 023150 | Mannopeptimycin (22 %) |
| 25 |  |  | Arylpolyene | 022720 | 022840 | Fusaricidin B (25 %) |
| 26 |  |  | NRPS | 020235 | 020385 | Ishigamide (61 %) |
| 27 |  |  | Lassopeptide | 015505 | 015535 | Moomysin (50 %) |
| 28 |  |  | Lantipeptide | 012240 | 012280 | SAL-2242 (77 %) |
| 29 |  |  | Terpene | 011020 | 011020 | Geosmin (100 %) |
| 30 |  |  | Ectoine | 009260 | 009275 | Ectoine (100 %) |
| 31 |  |  | Siderophore | 008850 | 008865 | Desferrioxamine E (100 %) |
| 32 |  |  | Siderophore | 008390 | 008415 | NA |
| 33 |  |  | Terpene | 005530 | 005555 | NA |
| 34 |  |  | Type I PKS – NRPS | 004155 | 004275 | Marinacarboline (23 %) |
| 35 |  |  | NRPS | 003585 | 003625 | Deimino-antipain (66 %) |
| 36 |  |  | NRPS like | 003060 | 003130 | NA |
| 37 |  |  | PKS type I | 002915 | 003030 | Tetronasin (9 %) |
| 38 |  |  | NRPS | 002830 | 002880 | Mannopeptimycin (14 %) |
| 39 |  |  | terpene | 002780 | 002800 | NA |
| 40 |  | X | Other-NRPS like | 002130 | 002230 | A83543A (8 %) |
| 41 |  | X | Butyrolactone | 001995 | 002040 | Cyphomycin (11 %) |
| 42 |  | X | PKS type I –NRPS | 001540 | 001635 | NA |
| 43 |  | X | NRPS | 001400 | 001445 | Teicoplanin (28 %) |
| 44 |  | X | nucleoside | 001190 | 001240 | Pseudouridimycin (68 %) |
| 45 |  | X | NRPS | 001130 | 001180 | NA |
| 46 |  | X | NRPS | 000480 | 000520 | NA |
| PLASMID | | | | | | |
| 1P |  |  | Type I PKS | 041090 | 041140 | Kanamycin (1 %) |
| 2P |  | X | NRPS | 040800 | 040830 | NA |

**Supplementary Table 2.** Genes from 145kb and 240kb deleted regions (marked genes belong to specific BGC) with NCBI protein ID and predicted function of the encoded protein

| Region | Locus_tag | BGC | Protein_ID | Predicted function |
| --- | --- | --- | --- | --- |
| Δ145 | SRIM_038560 | BGC 7 | QST85249.1 | iron ABC transporter permease CDS |
| Δ145 | SRIM_038565 | BGC 7 | QST85250.1 | iron ABC transporter permease CDS |
| Δ145 | SRIM_038570 | BGC 7 | QST85251.1 | iron-siderophore ABC transporter substrate-binding protein CDS |
| Δ145 | SRIM_038575 | BGC 7 | QST85252.1 | isochorismatase CDS |
| Δ145 | SRIM_038580 | BGC 7 | QST85253.1 | non-ribosomal peptide synthetase CDS |
| Δ145 | SRIM_038585 | BGC 7 | QST85254.1 | YncE family protein CDS |
| Δ145 | SRIM_038590 | BGC 7 | QST85255.1 | helix-turn-helix transcriptional regulator CDS |
| Δ145 | SRIM_038595 | BGC 7 | QST86478.1 | MFS transporter CDS |
| Δ145 | SRIM_038600 | BGC 7 | QST85256.1 | fes CDS |
| Δ145 | SRIM_038605 | BGC 7 | QST85257.1 | GNAT family N-acetyltransferase CDS |
| Δ145 | SRIM_038610 | BGC 7 | QST85258.1 | alpha/beta hydrolase CDS |
| Δ145 | SRIM_038615 | BGC 7 | QST85259.1 | AraC family transcriptional regulator CDS |
| Δ145 | SRIM_038620 | BGC 7 | QST86479.1 | class I SAM-dependent methyltransferase CDS |
| Δ145 | SRIM_038625 | BGC 7 | QST85260.1 | hypothetical protein CDS |
| Δ145 | SRIM_038630 | BGC 7 | QST85261.1 | iron chelate uptake ABC transporter family permease subunit CDS |
| Δ145 | SRIM_038635 | BGC 7 | QST86480.1 | iron chelate uptake ABC transporter family permease subunit CDS |
| Δ145 | SRIM_038640 | BGC 7 | QST85262.1 | dTDP-4-keto-6-deoxy-D-glucose epimerase CDS |
| Δ145 | SRIM_038645 | BGC 7 | QST85263.1 | rfbD CDS |
| Δ145 | SRIM_038650 | BGC 7 | QST85264.1 | rfbB CDS |
| Δ145 | SRIM_038655 | BGC 7 | QST85265.1 | glucose-1-phosphate thymidylyltransferase CDS |
| Δ145 | SRIM_038660 | BGC 7 | QST85266.1 | ABC transporter ATP-binding protein CDS |
| Δ145 | SRIM_038665 | BGC 7 | QST86481.1 | DUF1205 domain-containing protein CDS |
| Δ145 | SRIM_038670 | BGC 7 | QST85267.1 | fes CDS |
| Δ145 | SRIM_038675 | BGC 7 | QST85268.1 | ABC transporter substrate-binding protein CDS |
| Δ145 | SRIM_038680 | BGC 7 | QST85269.1 | cytochrome P450 CDS |
| Δ145 | SRIM_038685 |  |  | hypothetical protein CDS |
| Δ145 | SRIM_038690 |  |  | resuscitation-promoting factor rpfE CDS |
| Δ145 | SRIM_038695 |  | QST85270.1 | M48 family metalloprotease CDS |
| Δ145 | SRIM_038700 |  | QST85271.1 | helix-turn-helix transcriptional regulator CDS |
| Δ145 | SRIM_038705 |  | QST85272.1 | 4-hydroxybenzoate 3-monooxygenase CDS |
| Δ145 | SRIM_038710 |  | QST85273.1 | RNA polymerase sigma-70 factor CDS |
| Δ145 | SRIM_038715 |  | QST85274.1 | carboxymuconolactone decarboxylase family protein CDS |
| Δ145 | SRIM_038720 |  | QST85275.1 | alpha/beta hydrolase CDS |
| Δ145 | SRIM_038725 |  | QST85276.1 | chitinase CDS |
| Δ145 | SRIM_038730 |  |  | XRE family transcriptional regulator CDS |
| Δ145 | SRIM_038735 |  | QST85277.1 | hypothetical protein CDS |
| Δ145 | SRIM_038740 |  | QST85278.1 | hypothetical protein CDS |
| Δ145 | SRIM_038745 | BGC 6 | QST85279.1 | GDP-mannose 4,6-dehydratase CDS |
| Δ145 | SRIM_038750 | BGC 6 | QST85280.1 | thioesterase CDS |
| Δ145 | SRIM_038755 | BGC 6 | QST85281.1 | AAA family ATPase CDS |
| Δ145 | SRIM_038760 | BGC 6 | QST86482.1 | AAA family ATPase CDS |
| Δ145 | SRIM_038765 | BGC 6 | QST85282.1 | AAA family ATPase CDS |
| Δ145 | SRIM_038770 | BGC 6 | QST86483.1 | helix-turn-helix transcriptional regulator CDS |
| Δ145 | SRIM_038775 | BGC 6 | QST85283.1 | type I polyketide synthase CDS |
| Δ145 | SRIM_038780 | BGC 6 | QST85284.1 | type I polyketide synthase CDS |
| Δ145 | SRIM_038785 | BGC 6 | QST85285.1 | SDR family NAD(P)-dependent oxidoreductase CDS |
| Δ145 | SRIM_038790 | BGC 6 | QST85286.1 | SDR family NAD(P)-dependent oxidoreductase CDS |
| Δ145 | SRIM_038795 | BGC 6 | QST85287.1 | tyrosine-protein phosphatase CDS |
| Δ145 | SRIM_038800 | BGC 6 | QST86484.1 | GMC family oxidoreductase CDS |
| Δ145 | SRIM_038805 | BGC 6 | QST85288.1 | glycosyltransferase family 1 protein CDS |
| Δ145 | SRIM_038810 | BGC 6 | QST85289.1 | DegT/DnrJ/EryC1/StrS family aminotransferase CDS |
| Δ145 | SRIM_038815 | BGC 6 | QST85290.1 | cytochrome P450 CDS |
| Δ145 | SRIM_038820 | BGC 6 | QST85291.1 | ferredoxin CDS |
| Δ145 | SRIM_038825 | BGC 6 | QST85292.1 | AMP-binding protein CDS |
| Δ145 | SRIM_038830 | BGC 6 | QST85293.1 | IS1182-like element ISSdi1 family transposase CDS |
| Δ145 | SRIM_038835 | BGC 6 | QST85294.1 | ccrA CDS |
| Δ145 | SRIM_038840 | BGC 6 | QST85295.1 | GNAT family N-acetyltransferase CDS |
| Δ145 | SRIM_038845 | BGC 6 | QST85296.1 | alpha/beta hydrolase CDS |
| Δ145 | SRIM_038850 | BGC 6 |  | cytochrome P450 CDS |
| Δ145 | SRIM_038855 | BGC 6 | QST85297.1 | MarR family transcriptional regulator CDS |
| Δ145 | SRIM_038860 | BGC 6 |  | gamma-glutamyl-gamma-aminobutyrate hydrolase family protein CDS |
| Δ145 | SRIM_038865 | BGC 6 | QST85298.1 | hypothetical protein CDS |
| Δ145 | SRIM_038870 | BGC 6 | QST85299.1 | DeoR/GlpR transcriptional regulator CDS |
| Δ240 | SRIM_038875 |  | QST85300.1 | sigma-70 family RNA polymerase sigma factor CDS |
| Δ240 | SRIM_038880 |  | QST86485.1 | hypothetical protein CDS |
| Δ240 | SRIM_038885 |  | QST85301.1 | polyprenyl synthetase family protein CDS |
| Δ240 | SRIM_038890 |  | QST86486.1 | DUF2236 domain-containing protein CDS |
| Δ240 | SRIM_038895 |  | QST85302.1 | hypothetical protein CDS |
| Δ240 | SRIM_038900 |  | QST85303.1 | hypothetical protein CDS |
| Δ240 | SRIM_038905 |  | QST85304.1 | ATP-binding protein CDS |
| Δ240 | SRIM_038910 |  |  | DNA-binding response regulator CDS |
| Δ240 | SRIM_038915 |  | QST86487.1 | nitronate monooxygenase CDS |
| Δ240 | SRIM_038920 |  | QST85305.1 | SDR family oxidoreductase CDS |
| Δ240 | SRIM_038925 |  | QST85306.1 | DUF4239 domain-containing protein CDS |
| Δ240 | SRIM_038930 |  | QST85307.1 | dienelactone hydrolase family protein CDS |
| Δ240 | SRIM_038935 |  | QST85308.1 | winged helix-turn-helix transcriptional regulator CDS |
| Δ240 | SRIM_038940 |  | QST86488.1 | CPBP family intramembrane metalloprotease CDS |
| Δ240 | SRIM_038945 |  | QST85309.1 | hypothetical protein CDS |
| Δ240 | SRIM_038950 |  | QST85310.1 | SHOCT domain-containing protein CDS |
| Δ240 | SRIM_038955 |  | QST85311.1 | DUF1269 domain-containing protein CDS |
| Δ240 | SRIM_038960 |  | QST85312.1 | histidine phosphatase family protein CDS |
| Δ240 | SRIM_038965 |  | QST85313.1 | polyphosphate kinase 2 family protein CDS |
| Δ240 | SRIM_038970 |  | QST85314.1 | HAD-IC family P-type ATPase CDS |
| Δ240 | SRIM_038975 |  | QST85315.1 | methyltransferase domain-containing protein CDS |
| Δ240 | SRIM_038980 |  | QST85316.1 | flavodoxin CDS |
| Δ240 | SRIM_038985 |  | QST85317.1 | CBS domain-containing protein CDS |
| Δ240 | SRIM_038990 |  | QST85318.1 | universal stress protein CDS |
| Δ240 | SRIM_038995 |  | QST85319.1 | GNAT family N-acetyltransferase CDS |
| Δ240 | SRIM_039000 |  | QST85320.1 | HAD family hydrolase CDS |
| Δ240 | SRIM_039005 |  | QST85321.1 | HypC/HybG/HupF family hydrogenase formation chaperone CDS |
| Δ240 | SRIM_039010 |  | QST85322.1 | hypF CDS |
| Δ240 | SRIM_039015 |  | QST85323.1 | hydrogenase maturation protease CDS |
| Δ240 | SRIM_039020 |  | QST85324.1 | universal stress protein CDS |
| Δ240 | SRIM_039025 |  | QST85325.1 | response regulator transcription factor CDS |
| Δ240 | SRIM_039030 |  | QST85326.1 | flavodoxin CDS |
| Δ240 | SRIM_039035 |  | QST85327.1 | hypE CDS |
| Δ240 | SRIM_039040 |  | QST85328.1 | hypD CDS |
| Δ240 | SRIM_039045 |  | QST86489.1 | CBS domain-containing protein CDS |
| Δ240 | SRIM_039050 |  | QST85329.1 | Ni/Fe hydrogenase subunit alpha CDS |
| Δ240 | SRIM_039055 |  | QST85330.1 | oxidoreductase CDS |
| Δ240 | SRIM_039060 |  | QST85331.1 | oxidoreductase CDS |
| Δ240 | SRIM_039065 |  | QST86490.1 | cyclic nucleotide-binding domain-containing protein CDS |
| Δ240 | SRIM_039070 |  | QST85332.1 | 4Fe-4S ferredoxin CDS |
| Δ240 | SRIM_039075 |  | QST86491.1 | ppk2 CDS |
| Δ240 | SRIM_039080 |  | QST85333.1 | beta-lactamase family protein CDS |
| Δ240 | SRIM_039085 |  | QST85334.1 | APC family permease CDS |
| Δ240 | SRIM_039090 |  | QST86492.1 | GAF domain-containing protein CDS |
| Δ240 | SRIM_039095 |  | QST85335.1 | hypothetical protein CDS |
| Δ240 | SRIM_039100 |  |  | substrate-binding domain-containing protein CDS |
| Δ240 | SRIM_039105 |  | QST85336.1 | NIPSNAP family protein CDS |
| Δ240 | SRIM_039110 |  | QST86493.1 | hypothetical protein CDS |
| Δ240 | SRIM_039115 |  | QST85337.1 | SDR family oxidoreductase CDS |
| Δ240 | SRIM_039120 | BGC 5 | QST85338.1 | cytochrome P450 CDS |
| Δ240 | SRIM_039125 | BGC 5 | QST85339.1 | type I polyketide synthase CDS |
| Δ240 | SRIM_039130 | BGC 5 | QST85340.1 | cytochrome P450 CDS |
| Δ240 | SRIM_039135 | BGC 5 | QST85341.1 | type I polyketide synthase CDS |
| Δ240 | SRIM_039140 | BGC 5 | QST85342.1 | type I polyketide synthase CDS |
| Δ240 | SRIM_039145 | BGC 5 | QST85343.1 | GNAT family N-acetyltransferase CDS |
| Δ240 | SRIM_039150 | BGC 5 | QST85344.1 | flavodoxin family protein CDS |
| Δ240 | SRIM_039155 | BGC 5 | QST85345.1 | hypothetical protein CDS |
| Δ240 | SRIM_039160 | BGC 5 |  | IS481 family transposase CDS |
| Δ240 | SRIM_039165 | BGC 5 | QST85346.1 | ATP-binding protein CDS |
| Δ240 | SRIM_039170 | BGC 5 | QST85347.1 | MerR family transcriptional regulator CDS |
| Δ240 | SRIM_039175 |  | QST85348.1 | transposase CDS |
| Δ240 | SRIM_039180 |  |  | hypothetical protein CDS |
| Δ240 | SRIM_039185 |  | QST85349.1 | hypothetical protein CDS |
| Δ240 | SRIM_039190 |  | QST85350.1 | cold-shock protein CDS |
| Δ240 | SRIM_039195 |  | QST85351.1 | hypothetical protein CDS |
| Δ240 | SRIM_039200 |  | QST85352.1 | SDR family NAD(P)-dependent oxidoreductase CDS |
| Δ240 | SRIM_039205 |  | QST85353.1 | TetR/AcrR family transcriptional regulator CDS |
| Δ240 | SRIM_039210 |  | QST86494.1 | DUF4357 domain-containing protein CDS |
| Δ240 | SRIM_039215 |  | QST85354.1 | hypothetical protein CDS |
| Δ240 | SRIM_039220 |  |  | FAD-dependent monooxygenase CDS |
| Δ240 | SRIM_039225 |  | QST86495.1 | hypothetical protein CDS |
| Δ240 | SRIM_039230 |  | QST85355.1 | hypothetical protein CDS |
| Δ240 | SRIM_039235 |  | QST85356.1 | aldehyde dehydrogenase family protein CDS |
| Δ240 | SRIM_039240 |  | QST85357.1 | GAF domain-containing protein CDS |
| Δ240 | SRIM_039245 |  | QST85358.1 | hypothetical protein CDS |
| Δ240 | SRIM_039250 |  | QST86496.1 | hypothetical protein CDS |
| Δ240 | SRIM_039255 |  | QST85359.1 | cholesterol esterase CDS |

**Supplementary Table 3.** Homology regions, used in creation of genome reductions and rimA deletion and nearby targeting gRNA sequences

| **Homology region** | **Size (bp)** | **Location in ATCC 109970** | **gRNA sequence (5-3)** |
| --- | --- | --- | --- |
| Δ145kb_UP | 1942 | 8915913 - 8949972 | CGTGTACACCAAGTTCGGCG |
| Δ240kb_UP | 2084 | 9046463 - 9044380 | CACTTCCTGGAGAAAGACCG |
| Large del_DOWN | 1925 | 8804637 - 8802713 | GCCCCCGTCGAGATCCCCGT |
| ΔrimA_UP | 1580 | 8936676 - 8938205 | GAACTGGTACGCGACTGCGT |
| ΔrimA_DOWN | 1614 | 8939739 - 8941301 | CAGCCCTGGCCCCAGGAGAG |

**Supplementary Table 4.** The expected size of PCR amplicon, number of tested and verified mutants for 145kb, 240kb and *rimA* deletions.

| Deletion size (kb) | Primer pair | Size of amplificon (bp) | Tested samples | Verified correct deletion |
| --- | --- | --- | --- | --- |
| 145 | cPCR_Δ145kb_UP_Fw/cPCR_DOWN_Rw | 2168 | 10/10* | 3/4 |
| 240 | cPCR_Δ240kb_UP_Fw/cPCR_DOWN_Rw | 2334 | 10 | 6 |
| 1,6 | cPCR_ ΔrimA_UP_Fw/cPCR_ ΔrimA_Rw | 1623 | 10 | 9 |

*ATCC 10970 ΔOTC strain

**Supplementary Table 5.** Analysis of mutations in two S. rimosus ATCC 10970 Δ145kb strains. NCBI number of two genes conferring mutations are designated in the table * and **)

| Mutation | Strain | Type of mutation | Location (on ATCC 10970 genome) | Mutation length | Gene affected |
| --- | --- | --- | --- | --- | --- |
| Mut_1 | **Δ145kb, Δ145kb-b** | C to A | 1981300 | 1bp | / |
| Mut_2 | **Δ145kb** | deletion - frameshift | 7593131 | 1bp | ABC transporter substrate-binding protein /***** |
| Mut_3 | **Δ145kb-b** | deletion - frameshift | 7951003-7951007 | 5bp | STAS domain-containing protein /****** |
|  | ***** | ****** |  |  |  |
| NCBI ID | WP_003981595.1 | WP_003984775.1 |  |  |  |

**Supplementary Table 6. Control of the normalization process and metabolic uniformity of analyzed strains:** Comparison of expression (TPM) of house-keeping genes *rpoB* and *gyrB* [32,33] and secondary metabolism-related genes *bldD* and *ftsZ* between ATCC 10970 and engineered strains at time points T1=24h and T2=50h. Conditional formatting is used to help visualise differences (blue- smaller values, white -larger values)

| T1 Sample/gene (TPM) | rpoB (SRIM_018645) | gyrB (SRIM_021090) | bldD (SRIM_006700) | ftsZ (SRIM_010400) |
| --- | --- | --- | --- | --- |
| ATCC_1 | 486,41 | 396,66 | 383,8 | 373,46 |
| ATCC_2 | 481,39 | 435,1 | 344,42 | 330,98 |
| ATCC_3 | 465,57 | 411,17 | 382,88 | 319,99 |
| ATCC Δ145kb_1 | 491,77 | 373,86 | 494,25 | 315,5 |
| ATCC Δ145kb_2 | 396,55 | 456,06 | 455,21 | 270,84 |
| ATCC Δ145kb_3 | 346,18 | 413,85 | 460,78 | 286,37 |
| ATCC Δ240kb_1 | 550,5 | 459,61 | 430,91 | 363,98 |
| ATCC Δ240kb_2 | 617,8 | 474,21 | 412,42 | 354,47 |
| ATCC Δ240kb_3 | 587,43 | 482,14 | 360,86 | 356,18 |
|  |  |  |  |  |
| T2 Sample/gene (TPM) | rpoB (SRIM_018645) | gyrB (SRIM_021090) | bldD (SRIM_006700) | ftsZ (SRIM_010400) |
| ATCC_1 | 505,77 | 301,57 | 460,4 | 454,88 |
| ATCC_2 | 473,37 | 224,44 | 471,16 | 405,41 |
| ATCC_3 | 491,43 | 262,4 | 491,39 | 473,45 |
| ATCC Δ145kb_1 | 402,56 | 294,11 | 599,62 | 380,12 |
| ATCC Δ145kb_2 | 394,52 | 284,98 | 613,54 | 366,02 |
| ATCC Δ145kb_3 | 403,21 | 273,31 | 697,34 | 354,65 |
| ATCC Δ240kb_1 | 415,36 | 385,12 | 438,08 | 374,7 |
| ATCC Δ240kb_2 | 463,55 | 409,13 | 399,06 | 413,9 |
| ATCC Δ240kb_3 | 446,38 | 401,79 | 475,03 | 427,58 |

**Supplementary Table 7.** Compounds identified by HR-MS analysis

| Compound | [M + H]^+^ | Experimental [Da] | Calculated  [Da] | Difference [ppm] |
| --- | --- | --- | --- | --- |
| rimocidins | | | | |
| rimocidin | C_39_H_62_NO_14_ | 768.4170 | 768.4170 | 0 |
| rimocidin B | C_39_H_62_N_2_O_13_ | 767.4331 | 767.4330 | 0.1 |
| CE-108 | C_37_H_58_NO_14_ | 740.3844 | 740.3857 | -1.8 |
| CE-108 B | C_37_H_59_N_2_O_13_ | 739.4037 | 739.4017 | 2.0 |
| rimocidin (27-etil) | C_38_H_60_NO_14_ | 754.4014 | 754.4014 | 0 |
| longicatenamycins | | | | |
| longicatenamycin B | C_35_H_52_ClN_8_O_9_ | 763.3546 | 763.3546 | 0 |
| longicatenamycin C  (D-Val ->Ile) | C_36_H_54_ClN_8_O_9_ | 777.3699 | 777.3702 | -0.4 |

**Details on HR-MS analysis.**

All the compounds were analysed in positive mode (unit resolution and HRMS). For the initial experiments positive Q1 mode was used, and interesting peaks were then subjected to MS/MS analysis to produce fragmentation spectra. The fragmentation patterns and HRMS measurements have confirmed the compounds in the Table S11.

Details on type of column, mobile phase and flow:

Column: Phenomenex, Kinetex 2.6 µm XB-C18 100A, 100x2.1mm
Mobile phases: A MQ + 0.1% FA; B ACN + 0.1% FA
Temperature: 30 °C
Flow: 0.3 ml/min
Gradient: mobile phase B
0 min                5%
15 min            90%
18 min            90%
18.1 min         5%
23 min            5%

**Supplementary Table 8.** Strains used in this study

| **Name** | **Description** | **Reference** |
| --- | --- | --- |
| *E. coli* DH10B | *Escherichia coli* for cloning procedures | [78] |
| *E. coli* ET12567/pUB307 | *Escherichia coli* for conjugal transfer | [79] |
| *Streptomyces rimosus* ATCC 10970 | ATCC strain | NCBI: txid1265868 |
| *Streptomyces rimosus* ATCC 10970 Δ*145kb* | ATCC strain with 145kb deletion | This study |
| *Streptomyces rimosus* ATCC 10970 Δ*240kb* | ATCC strain with Δ*240kb* deletion | This study |
| *Streptomyces rimosus* ATCC 10970 Δ*otc* | Deletion of *otc* gene cluster - *otc* mutant | [28] |
| *Streptomyces rimosus* ATCC 10970 Δ*otc* Δ*145kb* | *otc* mutant, deletion of *145kb* | This study |
| *Streptomyces rimosus* M4018 | Industrial OTC-producing strain | [23] |
| *Streptomyces rimosus* R6-500 | Industrial OTC-producing strain | [25] |

**Supplementary Table 9.** Plasmids used in this study

| **Name** | **Description** | **Reference** |
| --- | --- | --- |
| pREP_P1_cas9 | Base plasmid with JTU412-derived unstable replicon, *P1* promoter, codon optimized *cas9* and sgRNA scaffold | [31] |
| pREP_P1_cas9 *tsr* | +*tsr* resistance | [26] |
| pYAC-ΦC31-Ts-OTC | Integrative plasmid with entire OTC cluster and ΦC31 integrase | [28] |
| pRep_P1_cas9_Δ145kb | *cas9* carrying plasmid for 145kb deletion | This study |
| pRep_P1_cas9_Δ240kb | *cas9* carrying plasmid for 240kb deletion | This study |
| pRep_P1_cas9_ΔrimA | *cas9* carrying plasmid for *rimA* inactivation | This study |

**Supplementary Table 10.** Primers used in this study

| **PCR primer name** | **Nucleotide sequence 5'→3'** | **PCR product** | **Size (bp)** |
| --- | --- | --- | --- |
| gRNA_Fw | gacccgcatcgacctgtcgcag | Δ145kb_gRNA, Δ240kb_gRNA, ΔrimA_gRNA | 460 |
| gRNA_Rw | ctcgagtcaaagcttgcgcgcggatc |  |  |
| Large del_DOWN_Fw | tttaaaccttcctgctgtgggtgctc | Large del_DOWN | 1967 |
| Large del_DOWN_Rw | gtcggcgggacgctgatcgtgcggttcgaaactagtctggcatcgctgtcggaagtggcg |  |  |
| Δ145kb_UP_Fw | aatattcttaaggatccgcgcgcaagctttgactcgagcgaagacctgtcccgcgtatc | Δ145kb_UP | 2020 |
| Δ145kb_UP_Rw | gctccgggcggaagagcacccacagcaggaaggtttaaaacttcgaggccatcgtcacc |  |  |
| Stich_delete_*SpeI* | cacttccgacagcgatgccagactattcgaaccgcacgatcagcgtcccg | / | / |
| Δ240kb_UP_Fw | gaatattcttaaggatccgcgcgcaagctttgactcgaggttgtccgggtcgaccttgtc | Δ240kb_UP | 2161 |
| Δ240kb_UP_Rw | ctccgggcggaagagcacccacagcaggaaggtttaaaaagtgcgtccctgaggcaac |  |  |
| ΔrimA_UP_Fw | cttaaggatccgcgcgcaagctttgactcgagcgttcgcggacctgggcgc | ΔrimA_UP | 1580 |
| ΔrimA_UP_Rw | gaaggacgacactcctgccgcctcttcggccgtctccg |  |  |
| ΔrimA_DOWN_Fw | gacggccgaagaggcggcaggagtgtcgtccttcgggc | ΔrimA_DOWN | 1614 |
| ΔrimA_DOWN_Rw | tcctggtcggcgggacgctgatcgtgcggttcgaagtcctgttcggcgtcttcgagacc |  |  |
| cPCR_Δ145kb_Fw | gacgacgcctcccgtccgcag | Δ145kb genotype | 2168 |
| cPCR_Δ145kb_Rw | gcgggttctgagccggcctg |  |  |
| cPCR_Δ240kb_Fw | gaacggcaccgtgatcttctcgcc | Δ240kb genotype | 2334 |
| cPCR_Δ240kb_Rw | gcgggttctgagccggcctg |  |  |
| cPCR_ΔrimA_Fw | cgaggtgttcgagaccgcgtc | Δ*rimA* genotype | 1623 |
| cPCR_ΔrimA_Rw | gtgccggtgagcccgaagg |  |  |
